# Exploration of the Supraspinal Hypotheses about Spinal Cord Stimulation and Dorsal Root Ganglion Stimulation: A Systematic Review

**DOI:** 10.3390/jcm10132766

**Published:** 2021-06-23

**Authors:** Lisa Goudman, Sander De Groote, Bengt Linderoth, Ann De Smedt, Sam Eldabe, Rui V. Duarte, Maarten Moens

**Affiliations:** 1 Department of Neurosurgery, Universitair Ziekenhuis Brussel, Laarbeeklaan 101, 1090 Brussels, Belgium; sanderdegroote49@gmail.com (S.D.G.); mtmoens@gmail.com (M.M.); 2 Center for Neurosciences (C4N), Vrije Universiteit Brussel, Laarbeeklaan 103, 1090 Brussels, Belgium; Ann.DeSmedt@uzbrussel.be; 3 STIMULUS Consortium (reSearch and TeachIng neuroModULation Uz bruSsel), Universitair Ziekenhuis Brussels, Laarbeeklaan 101, 1090 Brussels, Belgium; 4 Pain in Motion Research Group (PAIN), Department of Physiotherapy, Human Physiology and Anatomy, Faculty of Physical Education & Physiotherapy, Vrije Universiteit Brussel, Laarbeeklaan 103, 1090 Brussels, Belgium; 5 Department of Clinical Neuroscience, Karolinska Institutet, 171 77 Stockholm, Sweden; bengt.linderoth@gmail.com; 6 Department of Physical Medicine and Rehabilitation, Universitair Ziekenhuis Brussel, Laarbeeklaan 101, 1090 Brussels, Belgium; 7 Pain Clinic, The James Cook University Hospital, Middlesbrough TS4 3BW, UK; seldabe@nhs.net; 8 Liverpool Reviews and Implementation Group, Department of Health Data Science, University of Liverpool, Liverpool L69 3BX, UK; rui.duarte@liverpool.ac.uk; 9 Department of Radiology, Universitair Ziekenhuis Brussel, Laarbeeklaan 101, 1090 Brussels, Belgium

**Keywords:** spinal cord stimulation, mechanism of action, supraspinal, systematic review

## Abstract

Despite the established efficacy and effectiveness of Spinal Cord Stimulation (SCS), there is still no consensus on the supraspinal mechanisms of action of this therapy. The purpose of this study was to systematically review previously raised hypotheses concerning supraspinal mechanisms of action of SCS based on human, animal and computational studies. Searches were conducted using four electronic databases (PubMed, EMBASE, SCOPUS and Web of Science), backward reference searching and consultation with experts. The study protocol was registered prior to initiation of the review process (PROSPERO CRD42020161531). A total of 54 publications were included, 21 of which were animal studies, and 33 were human studies. The supraspinal hypotheses (*n* = 69) identified from the included studies could be categorized into six groups concerning the proposed supraspinal hypothesis, namely descending pathways (*n* = 24); ascending medial pathway (*n* = 13); ascending lateral pathway (*n* = 10); affective/motivational influences (*n* = 8); spinal–cerebral (thalamic)-loop (*n* = 3) and miscellaneous (*n* = 11). Scientific support is provided for the hypotheses identified. Modulation of the descending nociceptive inhibitory pathways, medial and lateral pathways were the most frequently reported hypotheses about the supraspinal mechanisms of action of SCS. These hypotheses were mainly supported by studies with a high or moderate confidence in the body of evidence.

## 1. Introduction

Spinal cord stimulation (SCS) is an effective neuromodulation technique, used for the management of a variety of chronic pain conditions [1]. Since the first report in 1967 by Shealy and colleagues [2], SCS has been used worldwide. The Gate Control Theory provided the initial mechanism of action for SCS [3], stating that the transmission of nociceptive signals could be inhibited at the dorsal horn of the spinal cord by stimulation of large-diameter nerve fibers [4]. However, after sectioning of the dorsal column in animal models receiving SCS, rostral and caudal stimulations produced a comparable inhibition of neuropathic manifestations, suggesting that both supraspinal and segmental mechanisms are activated by SCS and that rostral and caudal stimulations may activate different synaptic circuitries and transmitter systems [5]. As such, besides the segmental operating mechanisms, a wide variety of supraspinal mechanisms has been proposed. A previous systematic review explored the existing neurophysiological and functional neuroimaging literature to collect all articles about the effects of SCS on brain activity [6]. Based on the available literature, the thalamus and anterior cingulate cortex were identified as potential mediators of the pain experience. Additionally, SCS appeared to have an inhibitory effect on somatosensory evoked potentials [6]. Nevertheless, it was not possible to draw conclusive evidence about the supraspinal mechanisms of actions. 

During the last decade, a wide variety of SCS paradigms have been applied in clinical practice. Several authors have investigated the different types of SCS stimulation (i.e., traditional, burst, high frequency, high dose SCS) and suggested hypothetical supraspinal mechanisms of action of SCS, separated by the specific stimulation paradigm [7,8,9,10]. In the present study, the goal was to further explore the supraspinal mechanisms of action of SCS by collecting stated hypotheses about the mechanisms of action of SCS, regardless of the specific stimulation paradigm. By focusing on proposed hypotheses about supraspinal contributions, instead of discussing neurophysiological/neuroimaging results, we expected to observe and provide a higher degree of uniformity among the results, with a broader point of view on these mechanisms by including both direct and indirect (e.g., experimental pain measurements) evidence. Therefore, the aim of this systematic review was to explore the hypotheses on supraspinal mechanisms of action of SCS and the scientific support for each hypothesis.

## 2. Materials and Methods

### 2.1. Protocol and Registration

This systematic review was reported according to the PRISMA statement (Preferred Reporting Items for Systematic Review and Meta-Analyses) [11]. The protocol for this review was registered a priori in PROSPERO under the registration number CRD42020161531.

### 2.2. Search Strategy

The search strategy was developed based on the input of all authors. The initial searches were conducted in four electronic databases: PubMed, EMBASE, SCOPUS and Web of Science, on 17 September 2019. The searches were updated on 16 December 2020. The search strategy was created according to the PICO (Population-Intervention-Comparison-Outcome) framework [12] to explore supraspinal hypotheses (O) about the working mechanisms of SCS (I). The component “Comparison” was not relevant for our research question and, therefore, not defined. The component “Population” was not restricted (i.e., human, animal and computational studies were allowed); therefore, this term was not defined either. The final search strategy was built by combining both free-text terms and MeSH terms. Within each part of the PICO question (i.e., within ‘Intervention’ and ‘Outcome’), the search terms were combined using the Boolean operator OR. Between the components, the Boolean operator AND was used. No additional search filters were applied. The complete search strategy for PubMed can be found in Supplementary Material, Supplemental Digital Content 1. After building the search string in PubMed, it was individually adapted for the other three databases. We also screened the reference lists of all relevant publications for additional papers (backwards reference searching). Additionally, international experts were contacted to identify additional potentially relevant studies that were not found via electronic database searches or backward reference searching. 

### 2.3. Eligibility Criteria

Both observational and experimental studies investigating supraspinal mechanisms of SCS were considered for inclusion in this systematic review. Studies in which SCS or dorsal root ganglion (DRG) stimulation was explored were eligible for inclusion. All studies that explored supraspinal mechanisms of SCS and stated a hypothesis about the working mechanisms were eligible. Studies that only explored supraspinal mechanisms without creating a working hypothesis were excluded from this systematic review. This study was not restricted to a certain population, meaning that both animal and human studies were included. Computational studies were also eligible. Full eligibility criteria are presented in Table 1. 

### 2.4. Study Selection

Following de-duplication, all retrieved articles were screened for their title and abstract by two reviewers independently (SDG, MM), using Rayyan online software [13]. Subsequently, two reviewers (SDG, LG) performed the full-text screening independently from each other. The percentage agreement was calculated to assess inter-rater reliability, using R Studio 1.2.5019 (R version 3.6). Discrepancies were discussed after each stage of the screening in a consensus meeting with both reviewers and a third independent reviewer (LG for abstract screening and MM for full text screening). 

### 2.5. Data Extraction

The data extraction form included the following items, which were determined a priori: author, year, country, study design, population, SCS stimulation parameters, SCS duration (for human studies), outcome measurements, main findings to support the supraspinal hypothesis and the supraspinal hypothesis. Data extraction was performed by the first reviewer (SDG) and checked for correctness by the second reviewer (LG). Any discrepancies were discussed in a consensus meeting with the third reviewer (MM). 

### 2.6. Risk of Bias Assessment and Confidence in the Body of Evidence

The internal validity, meaning the degree to which the design, conduct and analysis of a study avoids bias, and the overall risk-of-bias of the included studies was assessed using the approach recommended by the NTP’s Office of Health Assessment and Translation (OHAT). The OHAT risk-of-bias rating tool consists of a set of questions and provides detailed instructions on how to evaluate methodological rigor in both human and animal studies. As recommended by OHAT, methodological criteria are dependent on the study design. Nine criteria were applied for animal studies, eight for human controlled trials, seven for cohort studies, case-control studies and cross-sectional studies and five criteria for case series to evaluate selection bias, confounding bias, performance bias, attrition/exclusion bias, detection bias, selective reporting bias and other sources of bias. Two authors (SDG, LG) independently assessed the risk-of-bias criteria for all included studies according to the following ratings: “++” definitely low risk of bias, “+” probably low risk of bias, “−” probably high risk of bias, or “−−” definitely high risk of bias. Potential disagreements between the authors were discussed and resolved by consensus with a third reviewer (MM). Afterwards, the overall risk-of-bias for each individual study was assessed through the OHAT approach for categorizing each study into tiers. Study quality was rated according to a three-tier system (1st tier: high confidence in the reported results, 2nd tier: moderate confidence in the reported results or 3rd tier: low confidence in the reported results). OHAT suggests the definition of “key” risk-of-bias criteria, which are given the highest weight in determining the overall risk-of-bias. For animal studies, the following “key” risk-of-bias criteria were determined: (1) “Were experimental conditions identical across study groups?”, (2) “Can we be confident in the exposure characterization?” and (3) “Can we be confident in the outcome assessment?” [14]. For human studies, the following key questions were determined: (1) “Did the study design or analysis account for important confounding and modifying variables?”, (2) “Can we be confident in the exposure characterization?”, (3) “Can we be confident in the outcome assessment?” [15]. The remaining risk-of-bias criteria were given less weight. Placement of a study into one of three study quality categories (1st tier, 2nd tier or 3rd tier) was contingent on the rating of these three key risk-of-bias criteria and the proportions in the rating of the remaining criteria. 

Confidence in the body of evidence was evaluated using the NTP/OHAT framework [16], which relied on the GRADE approach [17]. An initial classification was provided to each article based on the study design, to address causality. Subsequently, each included article (i.e., each body of evidence) was subjected to a critical evaluation of factors that may downgrade the initial confidence rating (risk of bias, unexplained inconsistency, indirectness, imprecision and publication bias) or factors that may upgrade it (large magnitude of effects, dose–response, residual confounding, cross-population/study consistency). The final confidence rating consisted of four main descriptors: high (++++), moderate (+++), low (++) or very low (+), to denote the confidence rating in the body of evidence [16].

## 3. Results

### 3.1. Study Selection

**Figure 1:**
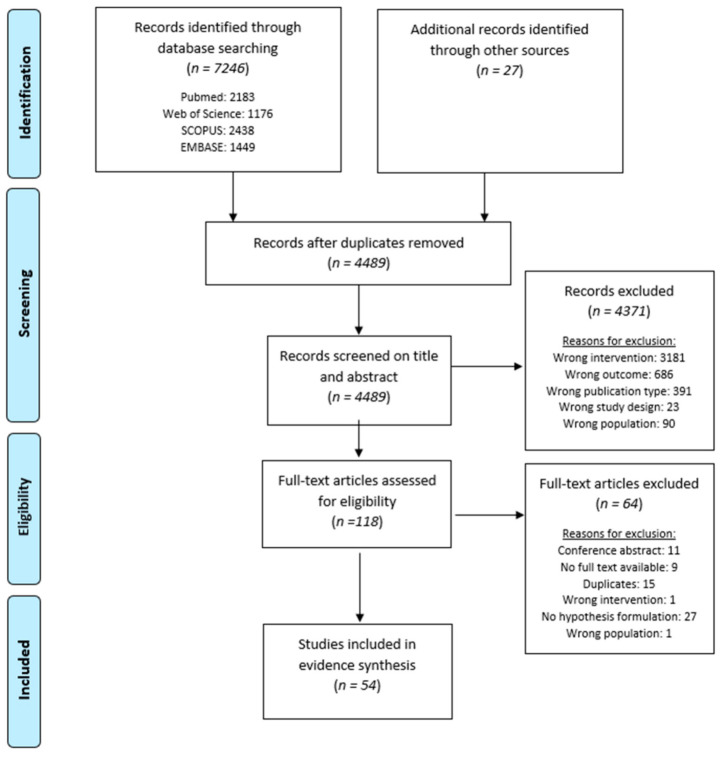
Flowchart of the systematic review. This figure shows the complete flowchart of the search and screening of articles for this systematic review. Abbreviations. *n*: number of studies.

The searches resulted in 4489 unique studies to be considered for screening, of which, eventually, 54 were eligible for inclusion in the systematic review (Figure 1). The percentage agreement between both reviewers for title and abstract screening and full text screening were 98.22% and 90.36%, respectively. The most prominent reasons for exclusion during the screening on title and abstract were wrong intervention (*n* = 3181), wrong outcome (*n* = 686) and wrong publication type (*n* = 391).

### 3.2. Study Characteristics

Thirty-three human studies and 21 animal studies were included in this systematic review. In terms of study design for human studies, 18 human controlled trials, 9 cohort studies and 6 case series were included. In total, 51 studies discussed SCS, and 3 studies discussed DRG stimulation. A complete overview of the characteristics of the included studies can be found in Supplementary Table S1. 

### 3.3. Risk of Bias Assessment

The results of the risk of bias assessment for each study can be found in Table 2 and Table 3. In animal studies, five of the reviewed studies were placed in the “1st tier”, 15 in the “2nd tier” and the remaining study was assigned to the “3rd tier”. Nine studies adequately addressed all three key risk-of-bias criteria. In the remaining 13 studies, methodological flaws in key criteria were mainly identified regarding two criteria: seven studies lacked information on confidence in the outcome assessment, identical experimental conditions were questionable in three studies and one study lacked information on both of them. Confidence in the exposure assessments was not assured in one study. A number of potential threats to the internal validity were also noticed in the remaining risk-of-bias criteria. Almost all studies lacked information on randomization exposure level, allocation concealment and blinding of research personnel. Finally, attrition or exclusion of outcome data was an item that was often insufficient. 

In human studies, 15 studies were allocated to the “1st tier”, and the remaining 18 studies were categorized as “2nd tier” studies. For the six case series that were included in this systematic review, all but one study scored low on the key risk-of-bias question concerning confounding and modifying variables. Two of those studies also scored low on confidence in the exposure characterization, and one study performed poorly on confidence in the outcome assessment. For human controlled trials (HCT’s) (*n* = 18), all studies scored high on the key risk-of-bias criteria for confidence in the exposure characterization, and all but three also scored high on confidence in outcome assessment. Eleven studies could be categorized as “1st tier”, while the remaining seven studies were allocated to “2nd tier”. From the eighteen HCT’s, ten studies lacked a clear randomization exposure level, nine studies provided insufficient information about blinding of research personnel, and three studies scored low on attrition or exclusion of outcome data. All but one study scored high on the item that all measured outcomes were reported, and none of the studies performed poorly on the criteria of other potential threats. Additionally, nine cohort studies were included in this systematic review, of which three could be assigned to the “1st tier” and the remaining six studies to the “2nd tier”. Only three studies had a high score on the key risk-of-bias criteria of confounding and modifying variables. The other key risk-of-bias criteria had a high score for all included studies, except for one study that lacked confidence in the outcome assessment. All other risk-of-bias criteria were appropriately reported, with room for improvement on attrition or exclusion of outcome data (insufficient for three studies). 

### 3.4. Results of Individual Studies

Data extraction resulted in 69 main supraspinal hypotheses being retrieved from the 54 included studies. These could be categorized into six groups concerning the proposed supraspinal hypothesis, namely (1) ascending medial pathway (*n* = 13), (2) ascending lateral pathway (*n* = 10), (3) descending pathway (*n* = 24), (4) affective/motivational influence (*n* = 8), (5) spinal–cerebral (thalamic)-loop (*n* = 3) and (6) miscellaneous (*n* = 11). 

#### 3.4.1. Ascending Pathways

Nociceptive input is transmitted from the spinal cord to the thalamus via a direct way (spinothalamic tract) or indirect way (spinoreticular, spinomesencephalic or mediolemniscal pathway) [71]. The hypotheses concerning supraspinal mechanisms of action focus on the spinothalamic tract with a distinction between the lateral spinothalamic tract, which ascends in the lateral column of the spinal cord, and the medial spinothalamic tract, ascending in the ventral column [71]. As such, SCS can be considered a bottom-up neuromodulation technique [18,51]. 

#### 3.4.2. Ascending Medial Pathway

The intralaminar nuclei, medial dorsal nucleus and midline nuclei of the thalamus are considered parts of the medial pain pathway. These nuclei receive multisynaptic tactile inputs. The medial dorsal nucleus projects to the prefrontal cortex and the insular cortex, including the prelimbic cortex, ventral and dorsal agranular insular cortex, lateral orbital cortex and anterior cingulate cortex [72]. The intralaminar nuclei project to the lateral cortex [73]. 

##### Scientific Support for This Hypothesis

Several authors hypothesized that SCS modulates the ascending medial pain pathway [26,28,40,45,46,47,49,51,57,58,59,70]. It has been demonstrated that SCS is able to provoke alterations in the activity and responsiveness of the intralaminar nuclei of the thalamus [49], medial parts of the thalamus [57,59], anterior cingulate cortex [26,28,46,47,59,70] and the dorsolateral prefrontal cortex [46,47]. An alteration in N2P2 amplitude by SCS [40] could be associated with an alteration in the ascending medial pathway, since this laser-evoked potential (LEP) component is presumably related to cingulate cortex inhibition, at least partly originating in the anterior cingulate cortex (ACC) [74]. Another study with EEG revealed an alteration in functional connectivity strength between FC3 and TP9 during SCS, which was suggested to be related to ACC activity [51]. Increased levels of gamma-aminobutyric acid (GABA) and decreased levels of glucose in the ipsilateral thalamus, as detected by 1H MR spectroscopy, could be explained by the activation of this paleospinothalamic pathway [58]. Finally, connectivity changes between regions of the salience, frontoparietal and central executive network after three months of SCS were denoted to a modulation of the medial pain pathway by SCS [45]. 

##### Confidence in the Body of Evidence for This Hypothesis

The hypothesis of the ascending medial pathway was supported by six (46.15%) studies that received a high confidence rating, five (38.46%) studies that received a moderate rating, one (7.69%) study with a low confidence rating and one (7.69%) study with very low confidence in the body of evidence. 

#### 3.4.3. Ascending Lateral Pathway

The lateral thalamic pain pathway includes the ventral posterior nuclei (VP) and posterior nuclei. The VP receives somatotopically organized tactile inputs from the mediolemniscal terminate, while the posterior nuclei receive input from roughly somatotopically organized extralemniscal ascending tactile inputs [71]. The VP connects with SI and SII reciprocally and topographically. The medial nuclei and triangular nuclei, which are both posterior nuclei, connect to SI/ SII and SII/posterior parts of the insular cortex, respectively [71,75]. 

##### Scientific Support for This Hypothesis

Several authors hypothesized that SCS modulates the ascending lateral pain pathway [26,27,28,40,46,47,54,67,70]. Evidence has been found for alterations in the ventral posterolateral nuclei of the thalamus [54], SI [26,28,46], SII [67] and posterior insula [47,67] through SCS. A study with laser-evoked potentials revealed an alteration of N1 by SCS [40], which is presumably generated in SI, SII and the insular cortex [76,77]. Additionally, DRG stimulation induced alterations in the ventral posteromedial and ventral posterolateral nuclei of the thalamus and the posterior nuclei of the thalamus [27]. 

##### Confidence in the Body of Evidence for This Hypothesis

The hypothesis of the ascending lateral pathway was supported by six (60.00%) studies that received a high confidence rating, three (30.00%) studies that received a moderate rating and one (10.00%) study with a low confidence in the body of the evidence. 

#### 3.4.4. Descending Pathways

In parallel to the ascending pathways, several descending control mechanisms operating, mainly executed through the periaqueductal gray matter (PAG)-rostroventromedial medulla axis [78]. Input to the PAG is delivered by several supraspinal structures, among which the prefrontal cortex, hypothalamus and amygdala contribute to the modulation of pain [79].

##### Scientific Support for This Hypothesis

It was suggested that SCS could stimulate at least five descending excitatory pathways with different conduction velocities [22]. Shimoji et al. suggested the activation of the dorsolateral funiculus and other descending tracts as a supraspinal mechanism of action of SCS [65]. Several studies denoted the influence of SCS on the descending serotonergic pathways [34,36] and the reduced GABA-mediated inhibition of PAG output neurons, leading to an increase of activity in the descending inhibitory pathways [24,35]. Others suggested that the therapeutic effect of SCS might rely on suppressing somatosensory processing [61]. Furthermore, it was suggested that SCS results in an elevated content of inhibitory neurotransmitters and a limited release of excitatory ones [68]. Another study with structural MRI revealed an increase in volume in the superior frontal white matter after three months of SCS, which could reflect an increase in the functioning of the descending pain inhibitory pathways [44]. Blair et al. suggested that SCS exerts an inhibitory influence on the conduction in multisynaptic extralemniscal pathways [39].

Several studies denoted the influence of SCS on brainstem loops and brainstem key regions [49], with contributions of the nucleus raphe magnus [25,31], the nucleus caudalis [19], rostroventromedial medulla [32] and locus coeruleus [33]. In relation to the descending pathways, changes in several supraspinal regions were found, namely in the orbitofrontal cortex [68] and anterior cingulate gyrus/cortex [46,68]. 

Two articles used an indirect evaluation through experimental pain measurements with Conditioned Pain Modulation (CPM). CPM is an indirect psychophysical measure to assess the functioning of the endogenous descending nociceptive inhibitory system, whereby SCS seemed to activate this descending system [50,66]. Additionally, sensory assessments with mechanical and thermal hypersensitivity indicated that SCS reduced hypersensitivity [5,23,29], indicative for the activation of brainstem structures in the descending inhibition. Similarly, DRG stimulation restored normal LEP physiology by reducing hyperactivity of the DRG neurons and subsequently reducing the influence of diffuse noxious inhibitory control over the second-order neurons [60]. Moreover, de Andrade et al. reported increased thresholds for sensorimotor reflexes during SCS, suggestive for the importance of complex cortical processing and descending inhibitory pathways [42]. 

##### Confidence in the Body of Evidence for This Hypothesis

The hypothesis of the descending pathways was supported by 10 (41.67%) studies that received a high confidence rating, eight (33.33%) studies that received a moderate rating, three (12.50%) studies with a low confidence and three (12.50%) studies with very low confidence in the evidence. 

#### 3.4.5. Spinal–Cerebral (Thalamic)-Loop

Three animal studies supported the hypothesis of a spinal–cerebral–spinal (thalamic) loop as a supraspinal mechanism of action of SCS [29,30,32]. This hypothesis was based on experiments with intracerebral neuroelectrical recordings in the rostro ventromedial medulla [32], spinal neuron recordings (after evoked spinal withdrawal reflexes) [30] and sensory assessments (behavioral tests) [29], all pointing to the direction of a spinal–supraspinal–spinal loop. 

##### Confidence in the Body of Evidence for This Hypothesis

The hypothesis of the spinal–cerebral (thalamic)-loop was supported by two (66.66%) studies that received a high confidence rating and one (33.33%) study with a low confidence in the evidence. 

#### 3.4.6. Affective/Motivational Influence

In total, seven articles mentioned the importance of affective and motivational processes to explain the effects of SCS [21,26,46,48,52,53,69,70]. In nine patients who responded well to chronic SCS within a two-year period, Weigel et al. denoted the involvement of cognitive and motivational processing during SCS [69]. In one of their experiments, De Ridder and Vanneste concluded that SCS stimulates the self-referential contextual (via the posterior cingulate cortex) aversive system (via the parahippocampus) [46]. Additionally, in 2019, the role of the dorsal ACC and posterior cingulate cortex was confirmed by Yearwood et al. who revealed an increase in metabolic rate (fluorodeoxyglucose positron emission tomography) in these structures during burst stimulation compared to tonic SCS [70]. Moreover, an activation (Positron emission tomography) in the ACC, dorsolateral prefrontal cortex and orbitofrontal cortex system during SCS was revealed in nine patients with a mixed etiology, whereby the authors hypothesized a key role for the thalamus in altering the pain threshold and sensory cognition [52]. The importance of affective–motivational aspects and sensory–discriminative dimensions was also revealed in the case series of Kunitake, with a decrease in regional blood flow in the parietal cortex contralateral to the painful side and an increase in the ACC and frontal cortex during SCS [53]. Two studies clearly indicated a key role for the limbic system in the effects of SCS [21,48]. More in detail, SCS is expected to reduce the affective processing by decreasing the connectivity strength between the somatosensory areas and the limbic areas [48]. Recently, a study with fMRI in rats, before, during and after SCS application, revealed that SCS is associated with the reward system, by an increase in BOLD signal levels in the nucleus accumbens and caudate putamen after SCS [26].

##### Confidence in the Body of Evidence for This Hypothesis

The hypothesis of the affective/motivational influence was supported by five (62.50%) studies that received a high confidence rating, two (25.00%) studies that received a moderate rating and one (12.50%) study with a low confidence in the evidence. 

#### 3.4.7. Miscellaneous 

Eleven articles described a supraspinal hypothesis that was not proposed as a main hypothesis in other articles [20,37,38,41,42,43,55,56,62,63,64]. Lind et al. targeted the human cerebrospinal fluid (CSF) in 14 SCS responsive neuropathic pain patients and revealed that 68 proteins were significantly altered when using SCS [55]. These proteins are involved in neuroprotection, nociceptive signaling, immune regulation and synaptic plasticity, suggesting that SCS triggers activity-dependent expression, metabolism or release of neuroplasticity-related genes in neurons and adjacent glial cells [55]. Similarly, Vallejo et al. also targeted gene expression, whereby the authors concluded that SCS may modulate immune and neuroprotective pathways, based on experiments on 84 rats [37]. Royds et al. performed lumbar punctures to evaluate T cell frequency, cytokines, chemokines, neurotrophins and a proteome analysis [63]. Alterations in the CSF proteome are predominately linked to synapse assembly and immune effectors. Additionally, due to the decreased expression of growth hormone A1, somatostatin and nucleobindin-2 after SCS, and given the fact that these are involved in hypothalamic functions, it may be suggested that SCS influences a supraspinal influence [63]. 

Bantli et al. conducted intracerebral neuroelectrical recordings in rhesus monkeys with electrodes in SI, SII and the parafascicular nucleus of the thalamus [20]. The reduction of amplitude of the long-latency components during SCS led to the hypothesis that interactions at the spinal or supraspinal level were responsible for the pain relief of SCS and not a conduction block of ascending pathways. More specifically, the authors hypothesized that patterns of convergence in projections from the spinal cord to the thalamus and secondary somatosensory cortex were responsible for the alterations induced by SCS [20]. Similarly, a study in a heterogenous group of patients with chronic pain used quantitative sensory testing (QST) to conclude that SCS has a central influence with spinal and/or supra-spinal contributions [38]. 

Two studies mentioned the function of SCS on regions of the pain matrix [41,56]. It was hypothesized that DRG stimulation could reverse the dysregulation that is induced by chronic pain in regions of the pain matrix [56]. Buentjen et al. suggested that SCS leads to a normalization of pathological spatiotemporal oscillatory patterns generated in the pain network, based on experiments with resting-state electroencephalography (EEG) [41].

Schlaier et al. used transcranial magnetic stimulation in five patients with chronic neuropathic pain, whereby they approved the idea that SCS modulates excitability and, probably, NMDA-related neuroplasticity at the supraspinal level [64]. More in detail, based on the augmentation of GABA-A- and GABA-B-mediated inhibitory mechanisms when reactivating SCS, the authors hypothesized that the thalamus might be an important mediator in the effect of SCS [64]. Another region that was denoted as an important region in relation to the effects of SCS is the hippocampus [43]. A voxel-based morphometry study in 11 patients with failed back surgery syndrome (FBSS) hypothesized that SCS can induce a normalization of the hippocampal function [43]. 

Polacek et al. investigated cortical somatosensory-evoked potentials in nine patients with FBSS, whereby it was revealed that SCS attenuates somatosensory processing in SI and SII, presumably resulting from a bombardment of the SI, SII and cingulate cortex by input from lemniscal neurons [62]. This heightened activity is expected to decrease the sensitivity to the allodynic component of neuropathic pain [62]. Finally, de Andrade et al. measured increased amplitude of sympathetic plantar skin responses, whereby SCS was able to reduce the sympathetic vasomotor activity and facilitate sympathetic sudomotor activity [42]. 

##### Confidence in the Body of Evidence for This Hypothesis

The hypotheses that were raised within this category received a high confidence rating (*n* = 6, 55.54%) or moderate (*n* = 5, 45.45%) confidence in the evidence.

## 4. Discussion

According to the International Association for the Study of Pain, pain is defined as “an unpleasant sensory and emotional experience associated with, or resembling that associated with, actual or potential tissue damage.” [80]. The key notes accompanying the newly revised definition clearly denote that pain is a personal experience that is influenced by biological, psychological and social factors [80], which suggests the complexity of pain. Due to the key role of the brain in creating this biopsychosocial concept, it seems straightforward that neuromodulation techniques also have an influence on supraspinal mechanisms [6]. Therefore, the aim of this study was to create a coherent idea about the supraspinal hypotheses of SCS and to provide a straightforward overview of all previously conducted experiments in this field. 

Based on this review, we were able to distil five main hypotheses and a miscellaneous group with several different hypotheses. Those relating to the descending pathways were by far the hypotheses that were raised by most articles, as the supraspinal mechanism of action of SCS (24/69, 34.78%), followed by the medial pathway (13/69, 18.8%), lateral pathway (10/69, 14.5%) and affective/motivational influence (8/69, 11.6%). The hypothesis of the descending pathway was supported by both animal and human studies, with results obtained from a broad range of experiments, which included experimental pain measurements, evoked potentials, intracerebral recordings and immunohistochemistry. Nevertheless, the contribution of the descending pathways is not unique to SCS. Transcutaneous electrical nerve stimulation is hypothesized to rely on activation of the periaqueductal gray and rostral ventromedial medulla, with an activation of the descending nociceptive inhibitory pathways [81]. Similarly, electroacupuncture is believed to alleviate pain via the mediation of these descending nociceptive inhibitory pathways [82]. Additionally, exercise-induced endogenous hypoalgesia (i.e., increased pain thresholds, pain tolerance and lower pain ratings during exercise) relies on the activation of brain-orchestrated descending nociceptive inhibition in response to exercise [83,84]. 

Several studies mentioned the importance of GABA in respect to the successful effects of SCS [24,35,58,64,85], where the results at the segmental and supraspinal level might seem contradictive at first sight. At the dorsal horn, closing the gate in the gate control theory is facilitated by inhibitory interneurons [86], whereby GABA is expected to play a pivotal role in this mechanism [87]. In SCS responders, SCS augments the release of GABA at the dorsal horn, thereby supporting the local inhibitory circuitry [88,89,90]. In the PAG, local GABAergic interneurons modulate the activity of output neurons that constitute the antinociceptive descending pathway [91]. During SCS, reduced GABA is observed (i.e., reduced GABA-mediated inhibition of PAG output neurons), leading to an increase of activity in the descending inhibitory pathways, with inhibitory functions on nociceptive transmission at spinal level [24,35] through GABA disinhibition in the descending PAG–RVM pathway [91]. Thus, reduced GABA in the PAG and increased GABA in the dorsal horn both support the inhibitory system. We mainly focused on GABA as the most common inhibitory substance in the central nervous system; however, in reality, a multitude of neurotransmitter systems are simultaneously involved in the effects of SCS [24]. 

Concerning the methodological quality of the articles, all hypotheses were supported by “1st tier” and “2nd tier” studies, except for the spinal–cerebral (thalamic)-loop hypothesis, which was supported by one “3rd tier” and two “2nd tier” studies. In animal studies, only 4 out of 21 studies (19.00%) scored properly on the first question of the risk of bias assessment, namely whether the exposure level was adequately randomized. The majority of the studies did not include a concurrent control group; therefore, this item scored poorly in this systematic review. Additionally, insufficient information was provided for blinding of the research personal and allocation concealment, leading to a lot of studies with a probable higher risk of bias. For human studies, controlled trials did not perform well on the item of whether the exposure level was adequately randomized, potentially leading to a risk of selection bias in these studies. Case series scored poorly on taking into account important confounding and modifying variables, whereby only one of them provided clear information on confounding variables and how to adjust for them in the analyses. Concerning the confidence in the body of evidence, all raised hypotheses were predominantly supported by studies with a high or moderate confidence. Even the studies that were categorized as miscellaneous were supported by studies with a high confidence in the evidence. 

In this systematic review, the raised supraspinal hypotheses of SCS and DRG stimulation were not associated with specific stimulation paradigms (standard SCS, high frequency SCS, burst SCS, high dose SCS, DRG stimulation). Previously, it has been suggested that burst SCS modulates the ascending lateral and medial pathways, with a higher modulation of the medial pathway compared to traditional SCS [46,47]. Nevertheless, in this review, the hypothesis of the ascending medial pathways was supported by several studies, in which several SCS paradigms (standard SCS, high frequency SCS) were used [45,49,58]. This leads to the suggestion that supraspinal hypotheses around the mechanism of action of SCS are intended to reflect the direct effects of applying electrical current to the dorsal columns, regardless of the specific stimulation paradigm. Nevertheless, it might be equally likely that the included studies evaluated indirect effects of obtaining pain relief. In the future, studies might focus on differentiating direct effects of applying SCS versus indirect effects by obtaining pain relief. It might be possible that the different SCS paradigms are distinguishable from each other in the way they contribute to direct and indirect effects of SCS. 

Similar to SCS, DRG stimulation might also have a contribution on the supraspinal structures [27,56,60]. Both in animal and human studies, DRG stimulation showed an effect on the ascending lateral pathways, pain matrix regions and descending modulatory pathways. This has been demonstrated with various measurement tools, including LEP, fMRI and 18 FDG PET/CT. This leads to the suggestion that both SCS and DRG stimulation rely on several shared supraspinal mechanisms. 

Finally, all hypotheses were supported by both human and animal studies, indicating that basic/fundamental and clinical research suggest similar hypotheses. No computational studies seem to have been identified through our systematic search. Computational studies can provide in-depth knowledge about neuromodulatory effects, which includes effects on axonal pathways, the optimization of SCS technologies and specific anatomical and technical aspects [92,93]. Presumably, further developments in this specific field will be able to further elucidate which supraspinal mechanisms are the most plausible.

## 5. Conclusions

Modulation of the descending nociceptive inhibitory pathways, followed by a modulation of the ascending medial and lateral pathways, respectively, were the most frequently reported hypotheses about the supraspinal mechanism of action of SCS, based on human and animal studies. These hypotheses were mainly supported by studies with a high or moderate confidence in the body of evidence. 

## Figures and Tables

**Table 1:** Eligibility criteria. Abbreviations. DRG: dorsal root ganglion, SCS: spinal cord stimulation.

Inclusion	Exclusion
Animals/humans treated with SCS or DRG stimulation. Computational studies about SCS/DRG were also allowed.	Other types of neuromodulation.
Supraspinal outcome measurements including, but not limited to, experimental pain measurements, brain imaging, histopathology.	No investigation of supraspinal outcome measurements.
Hypothesis about the supraspinal working mechanisms was explicitly stated in the article, including, but not limited to, expected theories, suggestions, hypotheses, assumptions or ideas.	No formulation of a possible underlying hypothesis.
Full-text (quasi) experimental or observational studies, case reports.	Systematic reviews and meta-analyses, narrative reviews, letters to the editor, conference abstracts, studies without available full-text version.
English, French or Dutch written.	Other languages.

**Table 2:** Risk of bias in individual animal studies.

Author/Year	Randomization Exposure Level	Allocation Concealment	Appropriate Comparison Groups	Confounding and Modifying Variables	Identical Experimental Cconditions	Blinding of Research Personnel	Attrition or Exclusion of Outcome Data	Confidence Exposure Characterization	Confidence Outcome Assessment	All Measured Outcomes Reported	Other Potential Threats	Quality Category (TIER-System)
Aguilar J. 2011 [18]	−	−			+	−	++	++	+	++	++	2nd tier
Atweh S.F. 1985 [19]	−−	NA			NA	NA	++	++	+	++	+	2nd tier
Bantli H. 1975 [20]	−−	−			+	−	−	−	+	−	−−	2nd tier
Barchini J. 2012 [5]	−	+			+	+	−	++	++	+	++	1st tier
Dejongste M. 1998 [21]	+	+			+	+	++	++	+	+	++	1st tier
Dembowsky K. 1985 [22]	−−	NA			+	−	++	+	+	−	++	2nd tier
El-Khoury C. 2002 [23]	−	−			+	−	−	++	−	++	++	2nd tier
Linderoth B. 1993 [24]	−−	NA			NA	−	−	+	+	++	++	2nd tier
Maeda Y. 2009 [25]	+	−			+	+	−	++	++	++	++	1st tier
Meuwissen K. 2020 [26]	+	+			+	−	++	++	+	++	++	1st tier
Pawela C. 2017 [27]	−	−			+	−	−	++	−	+	++	2nd tier
Quindlen-Hotek J. 2020 [28]	−	−			+	−	−	++	−	++	++	2nd tier
Saade N. 2015 [29]	−	−			+	−	−	++	−	+	++	2nd tier
Saade N. 1985 [30]	−−	NA			NA	NA	−	++	−	+	++	3th tier
Saade N. 1985 [31]	−−	NA			NA	NA	−	++	+	++	++	2nd tier
Song Z. 2013 [32]	−−	−			+	−	−	++	−	++	++	2nd tier
Song Z. 2013 [33]	−−	−			+	−	−	++	−	++	++	2nd tier
Song Z. 2009 [34]	−−	−			+	−	−	++	−	++	++	2nd tier
Stiller C. 1995 [35]	−	−			+	−	−	++	+	+	++	2nd tier
Tazawa T. 2015 [36]	−−	−			+	−	−	++	+	++	++	2nd tier
Vallejo R. 2019 [37]	+	−			+	+	+	++	++	++	++	1st tier

Key risk-of-bias criteria are indicated with a black frame. Definitely low risk of bias is indicated as ‘++’ and colored dark green, probably low risk of bias as ‘+’ and light green, probably high risk of bias as ‘−‘ and orange and definitely high risk of bias as ‘−−‘ and colored red. ‘NA’ stands for not applicable in this specific study. Depending on the exact study design, certain cells were not important and, therefore, not filled in.

**Table 3:** Risk of bias in individual human studies.

Author/Year	Design	Randomization Exposure level	Allocation Concealment	Appropriate Comparison Groups	Confounding and Modifying Variables	Identical Experimental Conditions	Blinding of Research Personnel and Subjects	Attrition or Exclusion of Outcome Data	Confidence Exposure Characterization	Confidence Outcome Assessment	All Measured Outcomes Reported	Other Potential Threats	Quality Category (TIER-System)
Ahmed S. 2015 [38]	HCT	−−	−				−	++	++	−	++	++	2nd tier
Blair R.D. 1975 [39]	case series				−				++	−	+	+	2nd tier
Bocci T. 2018 [40]	HCT	+	−				−	−	++	+	++	++	2nd tier
Buentjen L. 2020 [41]	HCT	−	−−				−	++	++	+	+	+	2nd tier
de Andrade DC 2010 [42]	HCT	+	+				+	++	++	+	++	++	1st tier
De Groote S. 2020 [43]	cohort			+	++			++	++	+	++	++	1st tier
De Groote S. 2020 [44]	cohort			+	++			++	++	+	++	++	1st tier
De Groote S. 2020 [45]	cohort			+	++			++	++	+	++	++	1st tier
De Ridder D. 2016 [46]	HCT	+	+				+	++	++	+	++	++	1st tier
De Ridder D. 2013 [47]	HCT	+	+				+	−	++	+	−	++	1st tier
Deogaonkar M. 2016 [48]	HCT	−	+				+	++	++	+	++	++	1st tier
Gildenberg P.L. 1980 [49]	case series				−				+	+	−−	+	2nd tier
Goudman L. 2019 [50]	cohort			+	−			++	++	−−	++	++	2nd tier
Goudman L. 2019 [51]	cohort			+	−			++	++	+	++	++	2nd tier
Kishima H. 2010 [52]	HCT	−−	NA				+	++	++	+	++	++	1st tier
Kunitake A. 2005 [53]	case series				++				++	+	+	++	1st tier
Larson S. 1974 [54]	HCT	−−	NA			−	−	−	++	+	+	+	2nd tier
Lind A.L. 2016 [55]	HCT	−−	+				+	+	++	+	++	++	1st tier
Mehta V. 2019 [56]	cohort			+	−			−	++	+	++	++	2nd tier
Modesti L.M. 1975 [57]	case series				−				++	+	+	+	2nd tier
Moens M. 2013 [58]	HCT	+	+				+	++	++	+	++	++	1st tier
Moens M. 2012 [59]	HCT	−	+				+	++	++	+	++	++	1st tier
Morgalla M.H. 2019 [60]	cohort			+	−			−	++	+	++	++	2nd tier
Pahapill P.A. 2014 [61]	case series				−				−	+	+	+	2nd tier
Polacek H. 2007 [62]	HCT	+	−				−	+	++	+	++	++	1st tier
Royds J. 2020 [63]	cohort			+	−			−	++	+	++	++	2nd tier
Schlaier J.R. 2007 [64]	HCT	−−	NA				−	++	++	+	++	++	2nd tier
Shimoji K. 1982 [65]	case series				−				−	+	++	+	2nd tier
Schuh-Hofer S. 2018 [66]	HCT	−	−				−	++	++	−−	++	++	2nd tier
Stancak A. 2008 [67]	HCT	+	−				−	++	++	+	++	++	1st tier
Sufianov A.A. 2014 [68]	cohort			+	−			++	++	+	++	++	2nd tier
Weigel R. 2015 [69]	HCT	−−	−				−	+	++	−	++	++	2nd tier
Yearwood T. 2019 [70]	HCT	++	++				+	++	++	+	++	++	1st tier

Key risk-of-bias criteria are indicated with a black frame. Definitely low risk of bias is indicated as ‘++’ and colored dark green, probably low risk of bias as ‘+’ and light green, probably high risk of bias as ‘−‘ and orange and definitely high risk of bias as ‘−−‘and colored red. ‘NA’ stands for not applicable in this specific study. Depending on the exact study design, certain cells were not important and, therefore, not filled in. Abbreviations. HCT: human controlled trial.

## Data Availability

All data is presented in the article and Supplementary Materials, no additional analysis were performed in this review.

## Supplementary Materials

The following are available online at https://www.mdpi.com/article/10.3390/jcm10132766/s1, Supplemental Digital Content 1: search terms. Supplementary Table S1: Characteristics of individual studies.
