# Supplementary material for: Exploration of the Supraspinal Hypotheses about Spinal Cord Stimulation and Dorsal Root Ganglion Stimulation: A Systematic Review
Source: J Clin Med. 2021 Jun 23;10(13):2766. doi: 10.3390/jcm10132766 (PMC8268298; doi:10.3390/jcm10132766)
Supplement: Supplementary file 1 [file jcm-10-02766-s001.zip › jcm-1235321-supplementary.pdf]

Journal of
Clinical Medicine

Supplementary Materials

Supplemental Digital Content 1

(((((((((((((((((("spinal cord stimulation") OR "neurostimulation") OR "neuromodulation") OR "dorsal column stimulation") OR "neurostimulator") OR "spinal cord stimulator") OR "dorsal column stimulator") OR "electrical stimulation") OR "pain stimulator") OR "pain stimulation") OR "tonic stimulation") OR "dorsal root ganglion stimulation") OR "high frequency stimulation") OR "burst stimulation") OR "spinal cord stimulation"[MeSH Terms]) OR "implantable neurostimulators"[MeSH Terms]) OR "electric stimulation therapy"[MeSH Terms])) AND (((((((((((((("supraspinal") OR "descending inhibitory pathway") OR "descending nociceptive inhibitory pathway") OR "descending pain pathway") OR "ascending pain pathway") OR "medial pain pathway") OR "lateral pain pathway") OR "mechanism of action") OR "pain loop") OR "pain matrix") OR "pain region") OR "pain regions") OR "pain brain region") OR "pain brain regions")

Supplementary Table S1. Characteristics of individual studies.

Author, year and country	Study design	Type of population/ source of animals	Type of stimulation	Duration	Outcome measurement	Main findings to support supraspinal hypothesis	Supraspinal hypothesis	Global supraspinal hypothesis	Confidence in the body of evidence
					**Animal studies**				
Aguilar et al., 2011, Spain [1]	Experimental animal study	Male Wistar rats (N=44) anodal sDCS: n= 16 cathodal sDCS: n=17 both types of sDCS: n=11	DCS in a dorso-ventral axis T9-T10 Amplitude: 1mA Duration: 15 minutes		**Neuroelectrical recording:** - spontaneous activity - Evoked activity in gracilis nucleus and somatosensory cortex after stimulation hindpaw.	**Anodal SCS:** - increase spontaneous activity nucleus gracilis and cortical activity - speed-up slow-wave oscillations - decrease amplitude local field potential nucleus gracilis. **Anodal SCS:** opposite effects.	Bottom-up neuromodulation technique	- ascending: medial pathway - ascending: lateral pathway	High
Atweh et al., 1985, Lebanon [2]	Experimental animal study	Adult male decorticate-decerebellate cats (N=10)	DCS Frequency: 300 Hz Pulse width: 0,1ms Amplitude: 0.1-0.5 mA (single pulses or short train of pulses)		**Spinal neuron recording:** Evoked activity Cingle cell activity in N. Caudatus	87% of nucleus caudalis neurons were inhibited by DC stimulation.	DCS inhibits nociceptive evoked activity of trigeminal neurons via brainstem pain-modulating centers (PAG/ raphe nuclei).	descending pathway	Moderate
Bantli et al., 1975, USA [3]	Experimental animal study	Adult rhesus monkeys with DCS transection at approximately T8	DCS at approximately T6.		**Intracerebral neuroelectrical recording:** Evoked potential with electrodes at the ventral posterolateral nucleus, parafascicular nucleas of the thalamus, SI and SII	Reduction of amplitude of long-latency component in SI, SII and parafascicular nucleus of the thalamus by DCS.	Interactions at spinal or supraspinal level are responsible for pain relief and not a conduction block of ascending pathways. More specifically, patterns of convergence in projections from spinal cord to posterior nuclear group and VPL of thalamus to secondary somatosensory cortex.	miscellaneous	Moderate
Barchini et al., 2012, Lebanon [4]	Experimental animal study	Adult female Sprague Dawley rats (N=38) with bilateral lesions cervical DC in 32 rats and intact spinal cord in 6 rats. Model weighing 250-300g	SCS *Rostral stimulation:* rostral to lesion *Caudal stimulation:* Low thoracic level Period of SCS: 5min. Frequency: 50 Hz Pulse width: 0,2 ms Amplitude: 0.25-0.7 mA or 2-4V		**Sensory assessment:** Mechanical sensitivity Heat sensitivity Cold sensitivity	SCS (both rostral and caudal) provides inhibition of neuropathic manifestations. Pre-treatment administration of receptor antagonists differentially influenced rostral and caudal SCS.	The supraspinal descending pain-modulating serotonergic, adrenergic and dopaminergic pathways are activated with SCS + involvement of the GABAergic- and catecholaminergic supraspinal mechanisms.	descending pathway	High

DeJongste et al., 1998, The Netherlands [5]	Experimental animal study	Male Wistar rats (N=20)<br>Model weighing ± 250 g<br><br>SCS: n=10<br>sham SCS: n=10	SCS at C7 and T2<br>Duration = 60 min.<br>Frequency = 80 Hz<br>Pulse width = 210 µsec<br>Amplitude = 8µA-40µA	**Immunocytochemistry:**<br>C-fos expression<br>HSP72-expression	After stimulation, increased C-fos expression was showed in PAG, nucleus tractus solitarius, dorsal motor nucleus of the vagus nerve, intermediolateral cell column, paraventricular hypothalamic nuclei, thalamus, amygdala and insular cortex.<br>HSP72 expression after SCS in the endothelium of the cerebral arteries of the entorhinal cortex, amygdala and hypothalamus, not in neurons.	- Long-term SCS effects due to alterations in limbic activity.<br>- Inhibition in intermediolateral cell column results from interneurons that modulate sensory inputs rather than from inhibition through supraspinal loop.	affective/motivational	High
Dembowsky et al., 1985, Germany [6]	Experimental animal study	Adult male and female cats (N=21)<br>intact neuraxis: n=14<br>spinalized at C2-3: n=7	SCS at T3-T4<br>Pulse width: 0.2-0.5 ms<br>Intensity: 1-20 V<br>Repetition rate stimulus every 0.9-3.9 s	**Spinal neuron recording:**<br>intracellular recording SPN at T3	Descending excitatory pathways onto SPNs: classification in 5 groups according to conduction velocity.	SCS could stimulates at least 5 descending exitatory pathways with different conduction velocities.	descending pathway	Low
El-Khoury et al., 2002, Lebanon [7]	Experimental animal study	Adult Sprague Dawley femal rats with mononeuropathy (N=35)<br>Model weighing 250-300 g<br><br>n=7 control group<br>2x n=7 SNI<br>2x n=7 CCI	DCS<br>Duration: 10-30 min.<br>Frequency: 75-100 HZ<br>Pulse width: 0,2 ms<br>Amplitudes: 0.25-0.7V or 1.5 to 4V	**Sensory assessment:**<br>Mechanical and cold allodynia<br>Heat hyperalgesia	SCS inhibits neuropathic manifestations.<br>(Depression of allodynia after DC lesions.)	Activation of the brainstem pain-modulating centers, via rostral inhibitory projections of the dorsal column nuclei.	descending pathway	High
Linderoth et al., 1993, Sweden [8]	Experimental animal study	Rats and cats	Standard SCS<br>Frequency: 50 or 100 Hz<br>Pulse width: 0.2 msec<br>Amplitude: 2/3 of motor threshold	**Microdialysis:**<br>dorsal horn and PAG microdialysis to explore GABA, serotonin and substance P neurotransmitter release	**GABA:**<br>Increased GABA in DH when SCS.<br>Decreased GABA in the ventrolateral PAG after SCS.<br>**SEROTONIN:**<br>Release of serotonin in the dorsal horn with SCS.<br>5-HT concentration in ventrolateral PAG does not change during SCS.<br>**SUBSTANCE P:**<br>No release of SP in the dorsal horn	GABAergic tonic inhibitory influence on neurons in descending tracts with inhibitory functions on nociceptive transmission at spinal level. Serotonin and substance p transmitter in descending inhibitory system.	descending pathway	Moderate

					or in the PAG following stimulation.			
Maeda et al., 2009, USA [9]	Experimental animal study	Adult Sprague-Dawley rats with SNI (N=37) Model weighing 250-350g<br><br>Control group without SNI: n=11	SCS upper lumbar region Duration: 30 min. Frequency: 4, 60, 100 Hz or sham Pulse width: 0.25 ms Amplitude: 0.16±0.02 mA or 0.42±0.03 mV	**<u>Immunohistochemistry:</u>** C-fos immunoreactivity in PAG, nucleus raphe magnus, DRG and spinal cord)	- 4Hz SCS: increase number of c-fos cells in NRM (5 minutes after stop SCS) in rats with SNI<br>- 4Hz and 60 Hz SCS: bilateral increase c-foss cells in dorsal horn of the cervical spine in SNI rats (2h after stop SCS).<br>- 100 Hz of SCS: no effect on the c-fos expression.	- Low frequency SCS: supraspinal + spinal mechanisms versus high frequency SCS spinal mechanisms.<br><br>- 4 Hz SCS: SCS produces a decrease in firing rate of ON cells and an increase in firing rate of OFF cells in the RVM, leading to inhibition of nociception.	descending pathway	High
Meuwissen et al., 2020, The Netherlands [10]	Experimental animal study	Male Sprague-Dawley rats Model weighing 150-200g<br><br>Partial Sciatic Nerve Ligation<br><br>N=17 whereby N=8 (standard SCS) and N=9 (burst SCS)	SCS at T13. Standard SCS (60min) : Frequency: 50 Hz Pulse width: 200 µsec Amplitude: 66 % MT<br><br>Biphasic Burst SCS (60min): Frequency = 40 Hz Burst mode 449Hz spike mode Pulse width = 1000µ sec Amplitude: 66 % MT	**<u>Sensory assessment:</u>** mechanical sensitivity before SCS, 30 min after and 60 min after SCS<br><br>**<u>fMRI:</u>** Measurements before, during, and after SCS (random allocation to burst or standard SCS)	Sensory assessment: No differences between burst and standard SCS.<br><br>fMRI:<br>- standard +burst SCS: increased BOLD signal in areas associated with location and intensity of pain, cognitive-emotional aspects. Stronger effects with burst SCS.<br>- burst SCS: also increased BOLD in reward system.	- Burst and standard: medial and lateral pathway but Burst stronger effect than standard on medial pathway.<br>- Burst also influences reward/motivational system.	- ascending: medial pathway<br>- ascending: lateral pathway<br>- affective/motivational	High
Pawela et al., 2017, USA [11]	Experimental animal study	Healthy male Sprague-Dawley rats Model weighing 250-350g<br><br>DRG stimulation: n=9<br>sham-surgery: n=7	DRG stimulation Frequency: 20Hz Pulse width: 150 µsec Amplitude: 1,2-1,5 mA	**<u>fMRI:</u>** Cerebral activation by BOLD after<br>- DRG stimulation<br>- noxious electrical stimuli	DRG stimulation attenuates BOLD signal response to noxious electrical hind paw stimulation	- Modulation neospinothalamic tract activity.<br>- DRG stim reduces painful input into thalamus	Ascending: lateral pathway	High
Quindlen-Hotek et al., 2020, USA [12]	Experimental animal study	Male Holtzman rats with NRC Model weighing 328-480g<br><br>C7 nerve root	*Standard SCS (2x1′) :* Frequency: 40 Hz Pulse width: 300 µsec Amplitude: 1.58±0.43 mA *Nonlinear Burst SCS (1′):* Frequency = 40 Hz Burst	**<u>Intracerebral neuroelectrical recording:</u>** Neuronal activity in ACC and S1 after SCS (3 different stimulation	Nonlinear burst SCS reduces firing in the ACC from a painful stimulus; 60% nonlinear burst also reduces firing in ACC during brush. Decreased firing in S1 neurons during light brush (standard SCS +	Burst and standard SCS: modulate the lateral pain pathway. Only Burst SCS modulates the medial pain pathway.	- Ascending: medial pathway<br>- Ascending: lateral pathway	High

		compression: n= 11<br>Sham operation: n= 6	mode<br>500Hz spike mode<br>Pulse width = 1000μ sec<br>Amplitude: 90 % MT<br>(0.60±0.22 mA)<br><br>60% MT (0.40±0.15 mA)	types) and light brush/ noxious pinch of the paw.	60% nonlinear burst) and pinch (60% nonlinear burst).			
Saade et al., 2015, Lebanon [13]	Experimental animal study	Adult Sprague - Dawley rats with SNI (and DLF lesions)<br>Model weighing 200-250g<br><br>SNI model (control group): n=5<br>SNI with DLF lesion: n = 30	Standard SCS<br>*Rostral DCNS:* level of obex<br>*Caudal SCS:* segment T11-12<br>Duration SCS: 5 min.<br>Frequency: 50 Hz<br>Pulse width: 0.2 ms<br>Amplitude: 0.25-0.7 mA or 2-4V	**Sensory assessment:**<br>Tactile sensitivity<br>Cold sensitivity<br>Heat sensitivity	- Decrease in thermal and tactile hypersensitivity after DCNS and SCS in SNI rats with and without DLF-lesion.<br>- GABA/serotonin/ adrenergic and dopamine receptor antagonists attenuated DCNS and SCS effects.	Inhibitory spinal-supraspinal-spinal loop with a mediating role for fibers running in the dosrolateral funiculi and ventrolateral funiculi.	- spinal-cerebral (thalamic)-loop<br>- descending pathway	High
Saade et al., 1985, Lebanon [14]	Experimental animal study	Cats with DC cuts on C1 and C4 (N=13)	SCS<br>1) DCS rostral to C1 DC cut<br>2) or stimulation to the RM.<br>Stimulation continuously with frequency: 100 Hz and pulse width: 0.2 ms or intermittently every 30 sec with 300 Hz and 30-100 ms duration.<br>Duration: 10 min.	**Spinal neuron recording:**<br>Spinal withdrawal flexion reflexes with electrodes at ventral root S1 or L7.	DCS and RM stimulation showed similar inhibition of the late discharge.<br>**Heat stimulation:**<br>Inhibition of the reflex discharge during continuously DCs.<br>**Electrical stimulation:**<br>The reflex discharge of 29 out of 33 motorneurons was inhibited by DCS	DCs activates a DC-brainstem-spinal loop.	spinal-cerebral (thalamic)-loop	Low
Saade et al., 1985, Lebanon [15]	Experimental animal study	Adult male cats with bilaterally DC cuts on C1 and C3 (N=18)	1) DCS rostral to C1 DC cut<br>2) stimulation to RM<br>Frequency: 300 Hz<br>Pulse width: 0.1 ms<br>Amplitude: 0.05-0.3 mA	**Spinal neuron recording:**<br>Electrodes in the dorsal horn L6-7	DCS inhibited the evoked activity of dorsal horn neurons (mainly in WDR neurons). Stimuli in the RM produced similar effects as DCs.	Brainstem loop, more specifically a DC-pontobulbar-spinal loop.	descending pathway	Low
Song et al., 2013, Sweden [16]	Experimental animal study	Male Sprague-Dawley rats with SNI Model weighing 250-350 g (N=34)<br><br>In case of mechanical	SCS at T11<br>Duration: 30 min.<br>Frequency: 50 Hz<br>Pulse width: 0.2 ms<br>Amplitude: 80% MT	**Intracerebral neuroelectrical recording:**<br>Neuronal activity in RVM	- Increase in the discharge rates of OFF-like and 5-HT-like cells during SCS in SCS-responder.<br>- Discharge rate of ON-cells and neutral cell were not affected during SCS.	SCS involves a spinal-supraspinal-spinal loop with activation of descending inhibitory conrols originating or relaying through RVM.	- spinal-cerebral (thalamic)-loop<br>- descending pathway	High

		hypersensitivity following SNI, SCS implantation:<br>N=30 SCS, of which 60% SCS responders<br>N=4 no SCS			- Anti-hypersensitivity effect of SCS was attenuated by $GABA_A$ receptor agonist, no effect of opioid receptor antagonist.			
Song et al., 2013, Sweden [17]	Experimental animal study	Male Sprague-Dawley rats with SNI model weighing 250-350 g (N=59)<br><br>In case of mechanical hypersensitivity following SNI, SCS implantation:<br>N=52 SCS, of which 60% SCS responders<br>N=7 no SCS	Monopolar SCS at the level of T11.<br>Duration: 30 min.<br>Frequency: 50 Hz<br>Pulse width: 0.2 ms<br>Amplitude: 80% MT	**Intracerebral neuroelectrical recording:**<br>Neuronal activity in LC<br>**ELISA:**<br>NA content	- increase in the discharge rates of LC neurons with SCS<br>- no change in the spinal NA content during SCS	Indirect contribution of LC neurons to descending pain-relieving effect by SCS through a LC-(PAG)-RVM loop instead of direct noradrenergic projections from LC to spinal cord.	descending pathway	High
Song et al.,2009, Sweden [18]	Experimental animal study	Male Sprague-Dawley rats with mononeuropathy Model weighing 250-350 g (N=96)<br><br>In case of tactile, cold hypersensitivity and heat hyperalgesia following SNI, SCS implantation:<br>N=62 SCS, of which 50% SCS responders<br>N=34 no SCS	SCS at T11<br>Duration of 30 min.<br>Frequency: 50 Hz<br>Pulse width: 0.2 ms<br>Amplitude: 80% MT	**Immunohistochemistry:**<br>Immunoreactivity (5-HT)<br>**ELISA:**<br>5-HT content	- SCS responders: increased 5-HT in dorsal quadrant of the ipsilateral spinal cord after SCS.<br>- bilateral increased 5-HT immunoreactivity in the dorsal horn (lamina I-II) after SCS.<br>- GABAb receptor antagonist enhanced the SCS effects on mechanical hypersensitivity.	Involvement of descending serotonergic pathways to inhibit spinal nociceptive transmission via a possible GABAergic link.	descending pathway	High
Stiller et al., 1995, Sweden [19]	Experimental animal study	Male Sprague-Dawley rats Model weighing 350-440g<br><br>Experiment 1: N=7<br>Experiment 2: SCS-group (N=7) +	Standard SCS<br>Duration: 2 x 30 min.<br>Frequency: 100 Hz<br>Pulse width: 0.2 msec.<br>Amplitude: 0.39-0.45 mA	**Microdialysis:**<br>GABA, glutamate, aspartate, Substance P and 5-HT concentrations with microdialysis in PAG	**Experiment 1:**<br>- SCS results in a decrease in GABA. No change in 5-HT and substance-P.<br>**Experiment 2:**<br>- After the second SCS session, decrease in GABA and glutamate concentration.	Reduced GABA-mediated inhibition of PAG output neurons leads to an increase of activity in descending inhibitory pathways.	descending pathway	High

		sham-stimulated group (N=6)						
Tazawa et al., 2015, Japan [20]	Experimental animal study	Male Sprague-Dawley spinal nerve-ligated rats, weighing 350-400 g.<br><br>In case of attenuation mechanical hypersensitivity after 1h SCS, eligible for true experiment.<br><br>Immunohistochemistry: SNL with SCS (N=4) + unstimulated SNL animals (N=4) + naïve rats (N=4).<br><br>Western blot analysis: SNL with SCS (N=6) + unstimulated SNL animals (N=6).	SCS at T10-T11 and L4-L5 level<br>Duration: 180 min.<br>Frequency: 50 Hz<br>Pulse width: 0.2 msec.<br>Amplitude: 70-80% MT		**<u>Immunohistochemistry:</u>** Immunofluorescent reactions; expression changes of synthetic enzymes of 5-HT in spinal dorsal horn.<br>**<u>Western blot analysis:</u>** 5-HT and norepinephrine expression in DRN and LC (TPH, DβH).<br>**<u>Sensory assessment:</u>** Mechanical sensitivity and hind-paw mechanical threshold	- SCS increased the number of serotonergic neurons in the DRN.<br>- No change in the noradrenergic neurons in the LC.<br>- Immunoreactivity ratio of DβH increased in the ipsilateral LC.<br>- SCS did not change the TPH and DβH expression in the spinal dorsal horn<br>- SCS reduces hypersensitivity in about 70% of the animals.	SCS influences supraspinal serotonergic pathway of the descending antinociceptive system with a smaller contribution of noradrenergic pathway.	Descending pathway	High
Vallejo et al., 2019, USA [21]	Experimental animal study	Male Sprague-Dawley rats (N=84) Model weighing 295 +/- 20g	Seven groups:<br>1) sham (N=7)<br>2) SNI injury (N=7)<br>3) injured rat + symmetric biphasic SCS (N=14)<br>4) injured rat + asymmetric biphasic SCS (N=14)<br>5) injured rat + asymmetric biphasic SCS (N=14)<br>6) injured rat + monophasic cathodic SCS (N=14)<br>7) injured rat + monophasic anodic SCS (N=14)	24h continuous stimulation	**<u>Sensory assessment:</u>** mechanical and cold thermal allodynia<br><br>**<u>Gene expression:</u>** Reverse-Transcriptase Polymerase Chain Reaction	Sensory assessment:<br>- no differences in cold sensitivity<br>- mechanical allodynia improved post-stim compared to pre-stim using cathodic, anodic, symmetric biphasic, or asymmetric biphasic 1:2 waveforms.<br><br>Gene expression:<br>- eight genes difference between pain versus SCS and between SCS forms.	SCS may modulate immune and neuroprotective pathways.	miscellaneous	High
				**Human studies**				

Ahmed et al., 2015, USA [22]	Controlled human trial	heterogeneous group of patients with chronic pain (N=19)		at least 4 weeks	**Experimental pain measurements:** - QST: warm sensation, heat pain threshold and heat pain tolerance.	- increase in QST parameters when SCS is on.	SCS has a central influence (spinal and/or supra-spinal).	Miscellaneous	High
Blair et al. 1975, Canada [23]	Case-series	Mixed etiologies (N=6): L4-5 root lesion: n=4 Spinal stenosis: n=1 Chronic pancreatitis: n=1	Standard DCS (T2-T3) Frequency: 9-250HZ Pulse width: 0.1-0.35 msec Voltage: 0-8V	Trial DCs; during first month after SCS implantation	**SSEP:** Tibial nerve stimulation	- DCS with frequency of 100 Hz decreases amplitude of the late SSEP components (peaks P4,N4 and P5). - DCs with greater stimulus intensity suppressed amplitude of all SSEP components.	SCS exerts an inhibitory influence on conduction in multisynaptic extralemniscal pathways.	descending pathway	Very low
Bocci et al., 2018, Italy [24]	Controlled human trial	Low back pain (N=30)	**Standard stimulation :** Frequency 10-200 Hz, pulse width 1-1000μs, amplitude 0.1-18 mA **High frequency stimulation:** Frequency 10 kHz, pulse width: 30μs, amplitude 0.1-13 mA **Theta-burst stimulation (TBS):** frequency: 40 Hz Burst mode, 500 Hz Spike mode, pulse width of 1000μs, amplitude not reported	> 3 months of SCS	**LEP** **Motor evoked potentials:** Cortical excitability: EMG recordings m. tibialis anterior	**LEP:** - High frequency SCS: reduction LEP N2P2 compared to baseline - TBS reduction N1 and N2N2 amplitude compared to LF and high frequency. - TBS increased N1 latency compared to baseline and LF. - TBS increased N2 latency compared to baseline. **Motor evoked potentials:** cSP duration was increased after TBS. Both high frequency SCS and TBS increases the ICF.	**Laser evoked potentials:** TBS has a predominant role to modulate both the sensory-discriminative (lateral pain pathway) and the affective-emotional dimension (medial pain pathway) of pain, subserved by the opercular cortex and the ACC. **Motor evoked potentials:** TBS could relief the pain by modulating both the intracortical glutamatergic and GABA(b)ergic networks.	- ascending: medial pathway - ascending: lateral pathway	High
Buentjen et al., 2020, Germany [25]	Controlled human trial	CRPS (N=2) Neuropathic pain syndrome (N=1) Deafferentation pain syndrome (N=1)	- OFF mode - Standard SCS (40Hz (N=3), 80 Hz (N=1)) - Burst SCS (200 Hz, burst rate of 40Hz, intensity 20% below paresthesia threshold) - High frequency SCS (1200 Hz, intensity 30% below paresthesia threshold) - low frequency SCS (2 Hz)	Measurements few days after SCS implantation	**Resting-state EEG:** Neuroelectrical recording	- SCS: reduction theta power (6-8 Hz). - Standard, high frequency and burst SCS: increase high-beta/low-gamma power (20–36 Hz). - Standard SCS: decrease high-gamma power (84–130 Hz).	Normalization of pathological spatiotemporal oscillatory patterns generated in the pain network.	miscellaneous	High

de Andrade et al., 2010, France [26]	Controlled human trial	FBSS (N=20), who successfully responded to SCS during 1 year.	SCS<br>Frequency: 50 Hz<br>Pulse width: 180 µsec<br>Voltage: 3V<br>in blocks of 3h<br>(monophasic square pulses in cycling mode)	1-13 years	**SSEP:**<br>Somatosensory system functioning to tibial nerve stimulation<br>**- sensorimotor reflexes with H- and RIII reflexes** at S1 territory.<br>**- Spinal motoneuron excitability** with F-waves Tibial nerve<br>**- Sympathetic skin responses (SSRs):** plantar	**SSEP:**<br>P40-SEP amplitude decreased during SCS.<br>**Sensorimotor reflexes:**<br>- increased thresholds during SCS<br>- increase latency RIII reflex during SCS<br>- H reflex amplitude decreased during SCS<br>**Spinal motoneuron excitability:**<br>- F-wave latency reduced during SCS<br>**SSR:**<br>increase SSR amplitude and decrease latency during SCS.	**Suprasegmental descending sensory pathways**<br>Complex cortical processing and descending inhibitory pathways are important in the modulation of spinal activities by SCS<br>**Sympathetic skin responses (SSRs)**<br>SCS reduce the sympathic vasomotor activities and facilitate sympathetic sudomotor activity.	- descending pathway<br>- miscellaneous	High
De Groote et al., 2020, Belgium [27]	Cohort study	FBSS (N=11)	High frequency SCS at 10 kHz at T8-T9:<br>Frequency: 10 kHz<br>Pulse width: 30 µsec<br>Amplitude: 1.5-2.5 mA	Three visits:<br>- before SCS<br>- 1 month of SCS<br>- 3 months SCS	**Structural MRI:**<br>VBM	Significant decrease in volume in left and right hippocampus.	SCS induces a normalisation of hippocampal function.	miscellaneous	Moderate
De Groote et al., 2020, Belgium [28]	Cohort study	FBSS (N=22)	Paresthesia-free SCS:<br>charge per pulse: 0.045 to 1.25 µC<br>charge per seconds: 350 to 750 µC/sec<br>duty cycle: 25–30%	Two visits:<br>- before SCS<br>- 3 months SCS	**Structural MRI**:<br>VBM	After 3 months of SCS, volume decrease and increase in several GM and WM areas.	The increase in volume in the superior frontal WM may reflect an increase in the functioning of the descending pain inhibitory pathways.	descending pathway	Moderate
De Groote et al., 2020, Belgium [29]	Cohort study	FBSS (N=10)	High frequency SCS at 10 kHz<br>Pulse width: 30µ sec<br>Frequency: 10 000 Hz<br>Amplitudes: 1.5 mA-2.5mA	Baseline (no SCS), 1 month of SCS and 3 months of SCS.	**rs-fMRI:**<br>rs-FC	After 3 months SCS, increased connectivity between anterior insula and regions of the frontoparietal network and the central executive network.	High frequency SCS at 10 kHz might influence salience network and therefore the medial pathway.	ascending: medial pathway	Moderate
De Ridder et al., 2016, Belgium [30]	Controlled human trial	FBSS (N=5)	**Burst SCS:**<br>Frequency:40 Hz Burst mode and 500Hz spike mode<br>Pulse width: 1000µ sec<br>90% paresthesia threshold	Trial SCS (minimally 28 days externalized stimulation) AND	**Resting-state EEG:**<br>Neuroelectrical recording	- Burst SCS activates the dACC, SI and the DLPFC more than standard SCS (alpha1 band).<br>- reduced pgACC/dACC ratio during burst SCS.<br>- Burst and standard SCS: Shared	Burst and standard SCS modulate<br>1) descending pain inhibitory system (via pgACC)<br>2) lateral pain pathways	- ascending: medial pathway<br>- descending pathway<br>- affective/motivanio	Moderate

			**Standard SCS:**<br>Frequency: 40 Hz standard mode<br>Pulse width: 330μ sec<br>Paresthesia coverage of painful area<br>**Sham SCS:**<br>stimulator off	3 weeks of randomized stimulation		activation in<br>A) theta band: SI, inferior parietal area, supramarginal gyrus, SII, the PCC and the parahippocampus.<br>B) gamma band: pgACC extending into vmPFC.	3) selfreferential contextual (via PCC) aversive system (via parahippocampus). Burst additionally modulates medial pain pathway by direct modulation spinothalamic pathways.	nal<br>- ascending: lateral pathway	
De Ridder et al., 2013, Belgium [31]	Controlled human trial	Eligible SCS patients (N=15) of which FBSS (N=5) were selected for EEG	**Burst SCS:**<br>Frequency:40 Hz Burst mode and 500Hz spike mode<br>Pulse width: 1000μ sec<br>90% paresthesia threshold<br>**Standard SCS:**<br>Frequency: 40 Hz standard mode<br>Pulse width: 330μ sec<br>Paresthesia coverage of painful area<br>**Sham SCS:**<br>stimulator off	Trial SCS (minimally 28 days externalized stimulation)<br>AND<br>3 weeks of randomized stimulation	**Resting-state EEG:**<br>Neuroelectrical recording	- Burst SCS activates the bilateral dACC, dLPFC more than standard SCS.<br>- Burst: Increased activity dACC and decreased activity parahippocampus compared to baseline and placebo.<br>- Standard SCS: deactivates the PCC and the posterior insula compared to placebo.	Burst SCS can modulates both the lateral pain pathway and the affective medial pain pathway.	- ascending: medial pathway<br>- ascending: lateral pathway	Moderate
Deogaonkar et al., 2015, USA [32]	Controlled human trial	N=10 in total;<br>FBSS: n=6<br>CRPS: n=3<br>Neuropathic pain: n=1	Standard SCS T6-T9<br>Parameters for optimal pain relief.	> 3 months of SCS	**rs-fMRI:**<br>rs-FC	Functional connectivity changes in many regions of the default mode and pain networks between SCS off and optimal SCS.	SCS reduces the affective processing by decreasing connectivity strength between the somatosensory areas and the limbic areas.	affective/motivational	High
Gildenberg et al.,1980, USA [33]	Case-series	N=2 in total;<br>postamputation pain: n=1<br>postlaminectomy arachnoiditis: n=1	DCS in the upper thoracic and lower cervical area.<br>Frequency: 20-40 Hz.		**Intrathalamic neuroelectrical recording:**<br>SSEPs with electrodes at the VPL and the interlaminar nucleus.	Short latency SEP in VPL not affected by DCS.<br>SCS modified long latency SEP component in intralaminar nucleus.	DCS could modulate the more multisynaptic paleospinothalamic system due to input from the pain pathways to the parafascicularis and intralaminar area. The brain stem is another important region at the supraspinal level during DCS.	- ascending: medial pathway<br>- descending pathway	Very low
Goudman et al., 2019,	Cohort study	FBSS (N=17)	**High frequency SCS at 10 kHz (N=8):**<br>Frequency: 10 kHz	**Baseline** = no SCS<br>**Second visit**	**Experimental pain measurements:**<br>- QST: EDT/EPT N.	- before SCS: less than 30% of patients CPM effect. Reactivation CPM with SCS.	SCS works through descending nociceptive inhibitory (serotonergic	descending pathway	Moderate

Belgium [34]			Pulse width: 30 µsec<br>Amplitude: 1.7-2.6 mA<br>**High dose SCS (N=9):**<br>Frequency: 500 Hz<br>Pulse width: 500 µsec<br>Amplitude: 1.2-3.7 V	= ± 3 months after implantation definitive SCS	Suralis<br>- CPM	- EDT increased with SCS, EPT only on non-symptomatic side.	and noradrenergic) pathways.<br>Additionally, a contribution of the midbrain, dorsal column, brainstem and bicortical structures is expected.		
Goudman et al., 2019, Belgium [35]	Cohort study	FBSS (N=8)	**Standard SCS:**<br>Frequency: 60 Hz<br>pulsewidth: 210 µsec<br>Amplitude: paresthesia covering painful areas<br>**High dose SCS:**<br>Frequency: 500 Hz<br>Pulsewidth: 500 µsec<br>Amplitude: 10% under level of paresthesia	**Baseline** = no SCS<br>**Second visit**= 76 days after implantation definitive SCS<br>**Third visit** = 176 days after implantation definitive SCS	**Resting-state EEG:**<br>Neuroelectrical recording	- Standard SCS lower average power spectrum than high dose SCS.<br>- FC3 to TP9 functional connectivity increase in beta band during High dose SCS.	In **high dose SCS**, the supraspinal effects might dominate through excitatory bottom-up pathways. High dose SCS could modulate the medial pain pathway by the affective-emotional dimension of pain of the ACC.<br>**Standard SCS** is expected more segmental effects as compared to High dose SCS. The reduced supraspinal effects could be explained through an inhibitory bottom-up effect based on the Gate Control Theory.	ascending: medial pathway	Moderate
Kishima et al., 2010, Japan [36]	Controlled human trial	Mixed etiologies (N=9);<br>FBSS: n=3<br>CRPS: n=2<br>cerebral hemorrhage: n=2<br>spinal infraction: n=1<br>SCI: n=1	Standard SCS<br>Frequency: 10-85 Hz<br>Pulse width: 210-450 µsec<br>Voltage: max. 10V<br>Duration of stimulation: 30 min.<br><br>OFF versus ON measurement	6 to 12 months	**H215O-PET:**<br>rCBF	SCS: increased rCBF in contralateral inferior parietal, DLPFC, contralateral ACC, ipsilateral precentral, contralateral thalamus, ipsilateral OFC, ipsilateral superior parietal.	SCS induces neuronal activity in contralateral thalamus -> pain relief -> thalamus alters pain threshold and sensory cognition.<br><br>SCS also activation ACC, DLPFC, OFC system (emotional).	affective/motivatio nal	High
Kunitake et al., 2005, Japan [37]	Case-series	Chronic neuropathic pain (N=11)	Standard SCS.<br>Upper limb pain: electrode at C3<br>Lower limb pain: electrode at T10	Trial SCS (n=6)<br>More than 1 Year SCS (n=5)	**SPECT scan:**<br>rCBF	**SCS responders:**<br>SCS causes increased rCBF was seen in the bilateral frontal cortex, the bACC and the cThal with SCS.	SCS influences both affective-motivational dimension of pain (frontal cortex, ACC, temporal cortex) as sensory-	affective/motivatio nal	Low

			Frequency: 5-25 Hz<br>Pulse width: 200 µsec<br>Amplitude: 0,5-1,5 V				discriminative dimension of pain processing (parietal cortex).		
Larson et al. 1974, USA [38]	Experimental human and animal trials	Human (N=18): Cancer pain, intractable pain<br><br>Animal (N=15): Stumptail macaque monkeys	Standard DCS<br>Frequency: 70-100 Hz<br>Pulse width: 0.25msec<br>Amplitude: 0.5-1mA	variable	**SSEP:**<br>scalp electrodes<br>**SSEP (animal):**<br>Somatosensory system functioning in SMC and VPL	- SCS decreases SSEP amplitudes of patients with cancer or intractable pain.<br>- SCS also decreases SSEP amplitudes recorded in the VPL and the SMC of monkeys.	The changes in activity could be mediated over extralemniscal pathways	ascending: lateral pathway	Low
Lind et al., 2016, Sweden [39]	Controlled human trial	Peripheral neuropathic pain (N=14)	Standard SCS.<br>Frequency: 50 Hz<br>No other parameters reported.	5 months -10 years (mean=4.4 years)	**Lumbar puncture:**<br>CSF levels of protein	Wide variety of proteins were altered between SCS on and off, which could be categorised under neuroprotection, synaptic plasticity, nociceptive signalling and immune regulation.	SCS triggers activity dependent expression, metabolism, or release of neuroplasticity related genes in spinal neurons and/or adjacent glial cells.	miscellaneous	Moderate
Mehta et al., 2019, UK [40]	Cohort study	Lumbar discogenic pain (N=9)	L2 DRG stimulation	Measurement before implantation and 4 weeks after stimulation	**18F FDG PET/CT:**<br>- metabolic activity	61 out of 85 RoI's of pain matrix revealed altered metabolic activity.	L2 DRG stimulation reverses the dysregulation in pain matrix regions areas caused by chronic pain.	miscellaneous	Moderate
Modesti et al., 1975, Italy [41]	Case-series	arteriosclerosis obliterans iliac artery (N=1)	Standard DCS at T1.<br>Frequency 65 Hz.<br>Voltage: 2.2V<br>Pulse width: 200 µsec<br>Duration: 5-60 sec.	4 months	**Intrathalamic neuroelectrical recording:**<br>Neuronal activity at the thalamus	DCS significantly decreases firing frequency of the thalamus.	Pain inhibition of the DCs due to the reduction in firing rate of units in the thalamus.	ascending: medial pathway	Low
Moens et al., 2013, Belgium [42]	Controlled human trial	FBSS (N=20)	Standard SCS at T9-10<br>Frequency: 60 Hz<br>Pulse width: 210µsec<br>Amplitude: 1,2-4,2 V	Trial SCS	**H-MR spectroscopy:**<br>Brain metabolite composition	- Increase of GABA concentration and decrease of glucose levels in the iThal after 9 minutes of SCS.<br>- During SCS: decrease glucose ipsilateral thalamus.	SCS: activation of the spino-reticulo-thalamic-cortical (paleospinothalamic) pathway.	ascending: medial pathway	High
Moens et al., 2012, Belgium [43]	Controlled human trial	FBSS (N=20)	Standard SCS at T9-10<br>Frequency: 60 Hz<br>Pulse width: 210µsec<br>Amplitude: 1,7-3,3 V	Trial SCS with outcome collection at 7-10 days postop and total trial 4 weeks	**fMRI:**<br>Cerebral activation by BOLD	Marked deactivation of the bilateral medial thalamus and its connections to the rostral and caudal cingulate cortex and the insula.	SCS inhibits a bilaterally distributed neural network, with a key role for the ipsilateral medial thalamus and his projections to the insula/ACC .The cerebello-thalamo-cortical circuit could work as an integration network of	ascending: medial pathway	High

							afferent signals for the modulation of pain.		
Morgalla et al, 2019, Germany [44]	Cohort study	Chronic neuropathic pain (N=12)	DRG stimulation at T12-L5 No parameters reported.	**Baseline** = no DRGS **Second visit**= 1 month of DRGS **Third visit** = six months of DRGS	**LEP:** Somatosensory system functioning	- P2 and N2P2 amplitudes increase after DRG at both time points.	DRG stimulation may normalize pain signal transfer from the periphery to supraspinal levels through the thermo-algesic pathways by restoring high-frequency AP filtering together with pain processing modulation (reducing influence of DNIC over second-order neurons) at the cortical level.	descending pathway	Moderate
Pahapill et al., 2014, USA [45]	Case-series	CRPS (N=2)	Patient 1: T10-T11 Patient 2: cervical SCS with Frequency: 85 Hz Pulse width: 360 μsec Amplitude: 1.3V		**MEG:** Evoked cortical activity	SCS normalize disorganization of hand D1/D5 cortical representation in SI. This result remains normalized when SCS was turned off for 30 minutes.	- SCS might rely on supressing somatosensory processing.	descending pathway	Very low
Polacek et al., 2007, Czech Republic [46]	Controlled human trial	FBSS (N=9)	Standard SCS (10 minutes in experiments).	12-43 months SCS	**SSEP:** Somatosensory system functioning after tibial and sural nerve stimulation	- SCS decreased SSEP amplitude in SI, bSII and in MC after tibial nerve stimulation. - SCS decreased amplitude in SI and bSII and increased amplitude in MC after sural nerve stimulation.	SCS: continuous input SI,SII and cingulate cortex by input from lemniscal neurons might lead to decreased somatosensory processing --> diminish sensitivity to neuropathic pain.	Miscellaneous	High
Royds et al., 2020, Ireland [47]	Cohort study	FNSS (N=1) FBSS (N=2) chronic post mastectomy pain (N=1) Only responders to burst SCS were included.	Burst SCS stimulation Burst rate: 40 Hz, intraburst rate 500 Hz, burst spike pulsewidth 1 ms, target amplitude 0.6 mA	measurement before implantation and after 8 weeks of SCS	**Lumbar puncture:** - T cell frequencies: flow cytometry - proteome analysis: mass spectrometry - cytokines, chemokines and neurotrophins: ELISA	- Alterations in the CSF proteome, predominately linked to synapse assembly and immune effectors. - no differences in frequency chemokines, cytokines and T cells.	Bursts SCS influences hypothalamic function.	miscellaneous	Moderate
Schlaier et al., 2007,	Controlled human trial	Neuropathic pain syndromes;	Standard SCS.	4-10 years	**TMS to stimulate C8 myotom**	- ICF increased during the "off" condition of SCS compared to the	- SCS modulates cortical excitability and supraspinal	miscellaneous	High

Germany [48]		lumbosacral root injury syndrome (N=5)			- Corticospinal excitability: RMT<br>- CSP to evaluate inhibitory GABA-B-mediated processes	"on" condition.<br>- RMT not modified.	NMDA related neuroplasticity.<br>- involvement of subcortical inhibitory processes at the level of the thalamus.		
Shimoji et al., 1982, Japan [49]	Case-series	Scoliosis (n=5)	Cervical DCS.	Trial SCS	**Evoked Spinal Cord Potentials:**<br>electrodes at the lumbar enlargement	DCS inhibits the N1 wave and facilitates the P2 wave.	The inhibition of the N1 wave probably reflect to orthodromic activation of the dorsolateral funiculus and other descending tracts.	descending pathway	Low
Schuh-Hofer et al.,2018, Germany [50]	Controlled human trial	Mixed etiologies (N=8) of which: Post-zoster Neuralgia: n=2<br>FBSS: n=2<br>Radiculopathy (of which one also post-zoster neuralgia): n=2<br>chronic mixed pain: n=1<br>Surgery of neuroma: n=1	Standard SCS<br>n= 2 SCS at cervical segment<br>n=6 SCS at lumbar segment<br><br>Frequency: 60-100 Hz<br>Pulse width: 100-400 µs<br>Voltage: 0.5-5V	1-11 years	**Experimental pain measurements:**<br>- QST: German research Network on Neuropathic Pain test battery<br>- CPM	- CPM reinforcement under SCS<br>- Reduction of enhanced temporal summation under SCS.	SCS could interact with descending pain-control pathways in the dorsolateral funiculus.	descending pathway	Moderate
Stancak et al., 2008, Czech Republic [51]	Controlled human trial	FBSS (N=8)	Standard SCS T9-T11<br>Frequency: between 45-85 Hz<br>Amplitude: between 0.6-5.0 mA	Trial SCS	**fMRI:**<br>Cerebral activation by BOLD	SCS increased BOLD signal in the SII, posterior insula and the mM1. A decrease in BOLD signal during SCS was observed in the bilateral primary motor cortices and SI.	- Acute pain relief by SCS: inhibiting dorsal-column nuclei or transmission in spino-thalamic tract neurons.<br>- Satiating neuronal circuits by stimulating dorsal columns so that there is a reduced input to the pain matrix.	ascending: lateral pathway	High
Sufianov et al., 2014, Russia [52]	Cohort study	Neuropathic pain syndrome (N=30) + 10 healthy persons as control	Standard SCS at T10-12.<br>Frequency: 40-120 Hz<br>Pulse witdh: 0,2-0,4 msec<br>Amplitude:1.5-6µV	**Baseline:**<br>Pre-implantation<br>**Second visit:**<br>3 months after implantation	**Resting-state EEG:**<br>Neuroelectrical recording<br>**18-FDG-PET/CT:**<br>Metabolic activity	**PET/CT:**<br>- partial normalization metabolic activity however 18F-FDG uptake in the OFC and ACG was still significantly increased compared to the healthy control group.<br><br>**EEG:**<br>- partial normalization EEG	SCS: Elevated content of inhibitory neurotransmitters and limited release of excitatory ones.	descending pathway	Moderate

						amplitudes delta and theta band, but still above healthy values.			
Weigel et al.,2015, Germany [53]	Controlled human trial	Chronic neuropathic pain (N=9); patients who responded well to chronic SCS within a two-year period.	Standard SCS (D9-D11).	≤ 2 years	**SSEP:** Somatosensory system functioning **Experimental pain measurements:** QST: EPT, EDT	**SSEPs** - stop SCS reduced amplitude N1,N2,N3. - P1 main source localized at mid cingulate region and more anterior during SCS. **QST** no difference in EDT, SCS decreased EPT at neuropathic pain side.	- Involvement of cognitive and motivational pain processing during SCS. - Block of peripheral pain pathways by antidromic AP to orthodromic activation of antinociceptive pathways of supraspinal systems [not based on own data]	affective/motivational	High
Yearwood et al, 2019, USA [54]	Controlled human trial	Chronic intractable pain of the trunk and/or limbs (N=7)	**Burst SCS:** Frequency: 40 Hz Burst mode and 500 Hz spike mode Pulse width: 1 msec Amplitude: patient perception **Standard SCS:** Frequency: 30-100 Hz Pulse width: 100-500 µsec Amplitude: comfortable paresthesia coverage	- Pre-implantation - 12 weeks of standard SCS - 12 weeks of burst SCS (cross-over)	**18-FDG-PET** Metabolic activity	- Burst SCS: increase metabolic rate dACC compared to baseline. - increase metabolic rate ACC and PCC and decrease metabolic rate sgACC in burst compared to standard SCS.	- Burst SCS stimulates dACC and PCC more than standard SCS. - Burst SCS: different pattern of brain activation in lateral and medial pathway compared to standard SCS.	- affective/ motivational - Ascending: medial pathway - Ascending: lateral pathway	Moderate

Abbreviations. ACC: anterior cingulate cortex, ACG: anterior cingulate gyrus, B : bilateral, BOLD: Blood oxygenation level dependent, c: contralateral, CCI: chronic constriction injury, CPM: conditioned pain modulation, CRPS: complex regional pain syndrome, CSF: cerebrospinal fluid, CSP: cortical silent period, D: dorsal, DβH : dopamine β-hydroxylase, DCNS: Dorsal column nuclei stimulation, DCS: dorsal column stimulation, DLF: Dorsolateral funiculi, DLPFC: dorsal lateral prefrontal cortex, DRG: dorsal root ganglion, DRN: dorsal raphe nucleus, EDT : electrical detection threshold, EEG: electroencephalography, ELISA: enzyme-linked immunosorbent assay, EMG: electromyography, EPT: electrical pain threshold, FBSS: failed back surgery syndrome, fMRI: functional magnetic resonance imaging, GABA: γ-aminobutyric acid, Hz: hertz, ICF : intracortical facilitation, i: ipsilateral, LC: locus coeruleus, LF : low frequency, LEP: laser evoked potentials, MC : mid-cingulate cortex, MEG: magneto-encephalography, MEP: motor evoked potentials, MT: motor threshold, N: nervus, NA: noradrenaline, OFC: orbitofrontal cortex, PAG : periaquaductorial gray, PCC: posterior cingulate cortex, PET/CT: positron-emission/computer tomography, pg: pregenual, QST: quantitative sensory testing, rCBF: regional cerebral blood flow, RM: raphe magnus nucleus, RMT: resting motor threshold, rs-FC: resting state functional connectivity, RVM: Rostral ventromedial medulla, SCI: spinal cord injury, SCS: spinal cord stimulation, SMC: sensory motor cortex, SNI: Spared nerve injury, SPECT: Single Photon Emission Computed Tomography, SSEP: somatosensory evoked potentials, SPN: sympathetic preganglionic neurones, SSR: sympathetic skin responses, S1 : primary somatosensory cortex, SII : secondary somatosensory cortex TBS: theta-burst stimulation, thal: thalamus, TMS: transcranial magnetic stimulation, TPH: tryptophan hydroxylase, VBM: voxel-based morphometry, vmPFC: ventromedial prefrontal cortex, VPL: nucleus ventralis posterior lateralis, WDR: wide dynamic range, WM: white matter, WT: withdrawal threshold, 5-HT: 5-hydroxytryptamine, 18F-FDG: 18F-fluorodeoxyglucose.

References

1. Aguilar, J.; Pulecchi, F.; Dilena, R.; Oliviero, A.; Priori, A.; Foffani, G. Spinal direct current stimulation modulates the activity of gracile nucleus and primary somatosensory cortex in anaesthetized rats. *J. Physiol.* **2011**, *589*, 4981–4996, doi:10.1113/jphysiol.2011.214189.
2. Atweh, S.F.; Dajani, B.M.; Saade, N.E.; Jabbur, S.J. Supraspinal inhibition of trigeminal input into subnucleus caudalis by dorsal column stimulation. *Brain Res.* **1985**, *348*, 401–404, doi:10.1016/0006-8993(85)90466-4.
3. Bantli, H.; Bloedel, J.R.; Thienprasit, P. Supraspinal interactions resulting from experimental dorsal column stimulation. *J. Neurosurg.* **1975**, *42*, 296–300, doi:10.3171/jns.1975.42.3.0296.
4. Barchini, J.; Tchachaghian, S.; Shamaa, F.; Jabbur, S.; Meyerson, B.; Song, Z.; Linderoth, B.; Saadé, N. Spinal segmental and supraspinal mechanisms underlying the pain-relieving effects of spinal cord stimulation: An experimental study in a rat model of neuropathy. *Neuroscience* **2012**, *215*, 196–208, doi:10.1016/j.neuroscience.2012.04.057.
5. Dejongste, M.J.; Hautvast, R.W.; Ruiters, M.H.; Ter Horst, G.J. Spinal Cord Stimulation and the Induction of c-fos and Heat Shock Protein 72 in the Central Nervous System of Rats. *Neuromodulation Technol. Neural Interface* **1998**, *1*, 73–84, doi:10.1111/j.1525-1403.1998.tb00020.x.
6. Dembowsky, K.; Czachurski, J.; Seller, H. An intracellular study of the synaptic input to sympathetic preganglionic neurones of the third thoracic segment of the cat. *J. Auton. Nerv. Syst.* **1985**, *13*, 201–244, doi:10.1016/0165-1838(85)90012-8.
7. El-Khoury, C.; Hawwa, N.; Baliki, M.; Atweh, S.; Jabbur, S.; Saadé, N. Attenuation of neuropathic pain by segmental and supraspinal activation of the dorsal column system in awake rats. *Neuroscience* **2002**, *112*, 541–553, doi:10.1016/s0306-4522(02)00111-2.
8. Linderoth, B.; Stiller, C.-O.; Gunasekera, L.; O'Connor, W.; Franck, J.; Gazelius, B.; Brodin, E. Release of Neurotransmitters in the CNS by Spinal Cord Stimulation: Survey of Present State of Knowledge and Recent Experimental Studies. *Ster. Funct. Neurosurg.* **1993**, *61*, 157–170, doi:10.1159/000100634.
9. Maeda, Y.; Ikeuchi, M.; Wacnik, P.; Sluka, K.A. Increased c-fos immunoreactivity in the spinal cord and brain following spinal cord stimulation is frequency-dependent. *Brain Res.* **2009**, *1259*, 40–50, doi:10.1016/j.brainres.2008.12.060.
10. Meuwissen, K.P.; Van Der Toorn, A.; Gu, J.W.; Zhang, T.C.; Dijkhuizen, R.M.; Joosten, E.A. Active Recharge Burst and Tonic Spinal Cord Stimulation Engage Different Supraspinal Mechanisms: A Functional Magnetic Resonance Imaging Study in Peripherally Injured Chronic Neuropathic Rats. *Pain Pr.* **2020**, *20*, 510–521, doi:10.1111/papr.12879.
11. Pawela, C.P.; Kramer, J.M.; Hogan, Q.H. Dorsal root ganglion stimulation attenuates the BOLD signal response to noxious sensory input in specific brain regions: Insights into a possible mechanism for analgesia. *NeuroImage* **2017**, *147*, 10–18, doi:10.1016/j.neuroimage.2016.11.046.
12. Quindlen-Hotek, J.C.; Kent, A.R.; De Anda, P.; Kartha, S.; Benison, A.M.; Winkelstein, B.A. Changes in Neuronal Activity in the Anterior Cingulate Cortex and Primary Somatosensory Cortex With Nonlinear Burst and Tonic Spinal Cord Stimulation. *Neuromodulation Technol. Neural Interface* **2020**, *23*, 594–604, doi:10.1111/ner.13116.
13. Saadé, N.E.; Barchini, J.; Tchachaghian, S.; Chamaa, F.; Jabbur, S.J.; Song, Z.; Meyerson, B.A.; Linderoth, B. The role of the dorsolateral funiculi in the pain relieving effect of spinal cord stimulation: A study in a rat model of neuropathic pain. *Exp. Brain Res.* **2014**, *233*, 1041–1052, doi:10.1007/s00221-014-4180-x.
14. Saadé, N.E.; Atweh, S.F.; Tabet, M.S.; Jabbur, S.J. Inhibition of nociceptive withdrawal flexion reflexes through a dorsal column-brainstem-spinal loop. *Brain Res.* **1985**, *335*, 306–308, doi:10.1016/0006-8993(85)90482-2.
15. Saade, N.E.; Tabet, M.S.; Banna, N.R.; Atweh, S.F.; Jabbur, S.J. Inhibition of nociceptive evoked activity in spinal neurons through a dorsal column-brainstem-spinal loop. *Brain Res.* **1985**, *339*, 115–118, doi:10.1016/0006-8993(85)90627-4.
16. Song, Z.; Ansah, O.; Meyerson, B.; Pertovaara, A.; Linderoth, B. The rostroventromedial medulla is engaged in the effects of spinal cord stimulation in a rodent model of neuropathic pain. *Neuroscience* **2013**, *247*, 134–144, doi:10.1016/j.neuroscience.2013.05.027.
17. Song, Z.; Ansah, O.; Meyerson, B.; Pertovaara, A.; Linderoth, B. Exploration of supraspinal mechanisms in effects of spinal cord stimulation: Role of the locus coeruleus. *Neuroscience* **2013**, *253*, 426–434, doi:10.1016/j.neuroscience.2013.09.006.
18. Song, Z.; Ultenius, C.; Meyerson, B.A.; Linderoth, B. Pain relief by spinal cord stimulation involves serotonergic mechanisms: An experimental study in a rat model of mononeuropathy. *Pain* **2009**, *147*, 241–248, doi:10.1016/j.pain.2009.09.020.
19. Stiller, C.-O.; Linderoth, B.; O'Connor, W.T.; Franck, J.; Falkenberg, T.; Ungerstedt, U.; Brodin, E. Repeated spinal cord stimulation decreases the extracellular level of γ-aminobutyric acid in the periaqueductal gray matter of freely moving rats. *Brain Res.* **1995**, *699*, 231–241, doi:10.1016/0006-8993(95)00911-9.
20. Tazawa, T.; Kamiya, Y.; Kobayashi, A.; Saeki, K.; Takiguchi, M.; Nakahashi, Y.; Shinbori, H.; Funakoshi, K.; Goto, T. Spinal Cord Stimulation Modulates Supraspinal Centers of the Descending Antinociceptive System in Rats with Unilateral Spinal Nerve Injury. *Mol. Pain* **2015**, *11*, 36, doi:10.1186/s12990-015-0039-9.
21. Vallejo, R.; Gupta, A.; Kelley, C.A.; Vallejo, A.; Rink, J.; Williams, J.M.; Cass, C.L.; Smith, W.J.; Benyamin, R.; Cedeño, D.L. Effects of Phase Polarity and Charge Balance Spinal Cord Stimulation on Behavior and Gene Expression in a Rat Model of Neuropathic Pain. *Neuromodulation Technol. Neural Interface* **2019**, *23*, 26–35, doi:10.1111/ner.12964.
22. Ahmed, S.U.; Zhang, Y.; Chen, L.; Hillary, K.S.; Cohen, A.; Vo, T.; Houghton, M.; Mao, J. Effects of Spinal Cord Stimulation on Pain Thresholds and Sensory Perceptions in Chronic Pain Patients. *Neuromodulation Technol. Neural Interface* **2015**, *18*, 355–360, doi:10.1111/ner.12316.

23. Blair, R.G.; Lee, R.G.; Vanderlinden, G. Dorsal Column Stimulation. Its effect on the somatosensory evoked response. *Arch. Neurol.* **1975**, *32*, 826–829, doi:10.1001/archneur.1975.00490540070009.
24. Bocci, T.; De Carolis, G.; Paroli, M.; Barloscio, D.; Parenti, L.; Tollapi, L.; Valeriani, M.; Sartucci, F. Neurophysiological Comparison Among Tonic, High Frequency, and Burst Spinal Cord Stimulation: Novel Insights Into Spinal and Brain Mechanisms of Action. *Neuromodulation Technol. Neural Interface* **2018**, *21*, 480–488, doi:10.1111/ner.12747.
25. Buentjen, L.; Vicheva, P.; Chander, B.; Beccard, S.; ScD, C.C.; Azañón, E.; Stenner, M.; Deliano, M. Spatial Filtering of Electroencephalography Reduces Artifacts and Enhances Signals Related to Spinal Cord Stimulation (SCS). *Neuromodulation Technol. Neural Interface* **2020**, doi:10.1111/ner.13266.
26. de Andrade, D.C.; Bendib, B.; Hattou, M.; Keravel, Y.; Nguyen, J.-P.; Lefaucheur, J.-P. Neurophysiological assessment of spinal cord stimulation in failed back surgery syndrome. *Pain* **2010**, *150*, 485–491, doi:10.1016/j.pain.2010.06.001.
27. De Groote, S.; Goudman, L.; Linderoth, B.; Buyck, F.; Rigoard, P.; De Jaeger, M.; Van Schuerbeek, P.; Peeters, R.; Sunaert, S.; Moens, M. A Regions of Interest Voxel-Based Morphometry Study of the Human Brain During High-Frequency Spinal Cord Stimulation in Patients with Failed Back Surgery Syndrome. *Pain Pr.* **2020**, *20*, 878–888, doi:10.1111/papr.12922.
28. De Groote, S.; Goudman, L.; Van Schuerbeek, P.; Peeters, R.; Sunaert, S.; Linderoth, B.; De Andrés, J.; Rigoard, P.; De Jaeger, M.; Moens, M. Effects of spinal cord stimulation on voxel-based brain morphometry in patients with failed back surgery syndrome. *Clin. Neurophysiol.* **2020**, *131*, 2578–2587, doi:10.1016/j.clinph.2020.07.024.
29. De Groote, S.; Goudman, L.; Peeters, R.; Linderoth, B.; Vanschuerbeek, P.; Sunaert, S.; Jaeger, M.D.; De Smedt, A.; Moens, M. Magnetic Resonance Imaging Exploration of the Human Brain During 10 kHz Spinal Cord Stimulation for Failed Back Surgery Syndrome: A Resting State Functional Magnetic Resonance Imaging Study. *Neuromodul. Technol. Neural Interface* **2019**, *23*, 46–55, doi:10.1111/ner.12954.
30. De Ridder, D.; Vanneste, S. Burst and Tonic Spinal Cord Stimulation: Different and Common Brain Mechanisms. *Neuromodulation Technol. Neural Interface* **2015**, *19*, 47–59, doi:10.1111/ner.12368.
31. De Ridder, D.; Plazier, M.; Kamerling, N.; Menovsky, T.; Vanneste, S. Burst Spinal Cord Stimulation for Limb and Back Pain. *World Neurosurg.* **2013**, *80*, 642–649.e1, doi:10.1016/j.wneu.2013.01.040.
32. Deogaonkar, M.; Sharma, M.; Oluigbo, C.; Nielson, D.M.; Yang, X.; Vera-Portocarrero, L.; Molnar, G.F.; Abduljalil, A.; Sederberg, P.B.; Knopp, M.; et al. Spinal Cord Stimulation (SCS) and Functional Magnetic Resonance Imaging (fMRI): Modulation of Cortical Connectivity With Therapeutic SCS. *Neuromodulation Technol. Neural Interface* **2015**, *19*, 142–153, doi:10.1111/ner.12346.
33. Gildenberg, P.; Murthy, K. Influence of Dorsal Column Stimulation upon Human Thalamic Somatosensory-Evoked Potentials. *Ster. Funct. Neurosurg.* **1980**, *43*, 8–17, doi:10.1159/000102228.
34. Goudman, L.; Brouns, R.; De Groote, S.; De Jaeger, M.; Huysmans, E.; Forget, P.; Moens, M. Association Between Spinal Cord Stimulation and Top-Down Nociceptive Inhibition in People with Failed Back Surgery Syndrome: A Cohort Study. *Phys. Ther.* **2019**, *99*, 915–923, doi:10.1093/ptj/pzz051.
35. Goudman, L.; Linderoth, B.; Nagels, G.; Huysmans, E.; Moens, M. Cortical Mapping in Conventional and High Dose Spinal Cord Stimulation: An Exploratory Power Spectrum and Functional Connectivity Analysis with Electroencephalography. *Neuromodulation Technol. Neural Interface* **2019**, *23*, 74–81, doi:10.1111/ner.12969.
36. Kishima, H.; Saitoh, Y.; Oshino, S.; Hosomi, K.; Ali, M.; Maruo, T.; Hirata, M.; Goto, T.; Yanagisawa, T.; Sumitani, M.; et al. Modulation of neuronal activity after spinal cord stimulation for neuropathic pain; H215O PET study. *NeuroImage* **2010**, *49*, 2564–2569, doi:10.1016/j.neuroimage.2009.10.054.
37. Kunitake, A.; Iwasaki, T.; Hidaka, N.; Nagamachi, S.; Katsuki, H.; Uno, T.; Takasaki, M. The effects of spinal cord stimulation on the neuronal activity of the brain in patients with chronic neuropathic pain. *Pain Res.* **2005**, *20*, 117–125, doi:10.11154/pain.20.117.
38. Larson, S.J.; Sances, A.; Riegel, D.H.; Meyer, G.A.; Dallmann, D.E.; Swiontek, T. Neurophysiological effects of dorsal column stimulation in man and monkey. *J. Neurosurg.* **1974**, *41*, 217–223, doi:10.3171/jns.1974.41.2.0217.
39. Lind, A.-L.; Khoonsari, P.E.; Sjödin, M.; Katila, L.; Wetterhall, M.; Gordh, T.; Kultima, K. Spinal Cord Stimulation Alters Protein Levels in the Cerebrospinal Fluid of Neuropathic Pain Patients: A Proteomic Mass Spectrometric Analysis. *Neuromodulation Technol. Neural Interface* **2016**, *19*, 549–562, doi:10.1111/ner.12473.
40. Mehta, V.; Bouchareb, Y.; Ramaswamy, S.; Ahmad, A.; Wodehouse, T.; Haroon, A. Metabolic Imaging of Pain Matrix Using 18 F Fluoro-deoxyglucose Positron Emission Tomography/Computed Tomography for Patients Undergoing L2 Dorsal Root Ganglion Stimulation for Low Back Pain. *Neuromodulation Technol. Neural Interface* **2019**, *23*, 222–233, doi:10.1111/ner.13095.
41. Modesti, L.M.; Waszak, M. Firing Pattern of Cells in Human Thalamus during Dorsal Column Stimulation. *Ster. Funct. Neurosurg.* **1975**, *38*, 251–258, doi:10.1159/000102667.
42. Moens, M.; Mariën, P.; Brouns, R.; Poelaert, J.; De Smedt, A.; Buyl, R.; Droogmans, S.; Van Schuerbeek, P.; Sunaert, S.; Nuttin, B. Spinal cord stimulation modulates cerebral neurobiology: A proton magnetic resonance spectroscopy study. *Neuroradiology* **2013**, *55*, 1039–1047, doi:10.1007/s00234-013-1200-7.
43. Moens, M.; Sunaert, S.; Mariën, P.; Brouns, R.; De Smedt, A.; Droogmans, S.; Van Schuerbeek, P.; Peeters, R.; Poelaert, J.; Nuttin, B. Spinal cord stimulation modulates cerebral function: An fMRI study. *Neuroradiology* **2012**, *54*, 1399–1407, doi:10.1007/s00234-012-1087-8.
44. Morgalla, M.H.; Filho, M.F.D.B.; Chander, B.S.; Soekadar, S.R.; Tatagiba, M.; Lepski, G. Neurophysiological Effects of Dorsal Root Ganglion Stimulation (DRGS) in Pain Processing at the Cortical Level. *Neuromodulation Technol. Neural Interface* **2018**, *22*, 36–43, doi:10.1111/ner.12900.

45. Pahapill, P.A.; Zhang, W. Restoration of Altered Somatosensory Cortical Representation with Spinal Cord Stimulation Therapy in a Patient with Complex Regional Pain Syndrome: A Magnetoencephalography Case Study. *Neuromodulation Technol. Neural Interface* **2014**, *17*, 22–27, doi:10.1111/ner.12033.
46. Poláček, H.; Kozák, J.; Vrba, I.; Vrána, J.; Stančák, A. Effects of spinal cord stimulation on the cortical somatosensory evoked potentials in failed back surgery syndrome patients. *Clin. Neurophysiol.* **2007**, *118*, 1291–1302, doi:10.1016/j.clinph.2007.02.029.
47. Royds, J.; Conroy, M.J.; Dunne, M.R.; Cassidy, H.; Matallanas, D.; Lysaght, J.; McCrory, C. Examination and characterisation of burst spinal cord stimulation on cerebrospinal fluid cellular and protein constituents in patient responders with chronic neuropathic pain—A Pilot Study. *J. Neuroimmunol.* **2020**, *344*, 577249, doi:10.1016/j.jneuroim.2020.577249.
48. Schlaier, J.R.; Eichhammer, P.; Langguth, B.; Doenitz, C.; Binder, H.; Hajak, G.; Brawanski, A. Effects of spinal cord stimulation on cortical excitability in patients with chronic neuropathic pain: A pilot study. *Eur. J. Pain* **2007**, *11*, 863–868, doi:10.1016/j.ejpain.2007.01.004.
49. Shimoji, K.; Shimizu, H.; Maruyama, Y.; Matsuki, M.; Kuribayashi, H.; Fujioka, H. Dorsal column stimulation in man: Facilitation of primary afferent depolarization. *Anesth. Analg.* **1982**, *61*, 410–413.
50. Schuh-Hofer, S.; Fischer, J.; Unterberg, A.; Treede, R.-D.; Ahmadi, R. Spinal cord stimulation modulates descending pain inhibition and temporal summation of pricking pain in patients with neuropathic pain. *Acta Neurochir.* **2018**, *160*, 2509–2519, doi:10.1007/s00701-018-3669-7.
51. Stančák, A.; Kozák, J.; Vrba, I.; Tintěra, J.; Vrána, J.; Poláček, H.; Stančák, M. Functional magnetic resonance imaging of cerebral activation during spinal cord stimulation in failed back surgery syndrome patients. *Eur. J. Pain* **2008**, *12*, 137–148, doi:10.1016/j.ejpain.2007.03.003.
52. Sufianov, A.A.; Shapkin, A.G.; Sufianova, G.Z.; Elishev, V.G.; Barashin, D.A.; Berdichevskii, V.B.; Churkin, S.V. Functional and Metabolic Changes in the Brain in Neuropathic Pain Syndrome against the Background of Chronic Epidural Electrostimulation of the Spinal Cord. *Bull. Exp. Biol. Med.* **2014**, *157*, 462–465, doi:10.1007/s10517-014-2591-0.
53. Weigel, R.; Capelle, H.H.; Flor, H.; Krauss, J.K. Event-related cortical processing in neuropathic pain under long-term spinal cord stimulation. *Pain Physician* **2015**, *18*, 185–194.
54. Yearwood, T.; De Ridder, D.; Bin Yoo, H.; Falowski, S.; Venkatesan, L.; To, W.T.; Vanneste, S. Comparison of Neural Activity in Chronic Pain Patients During Tonic and Burst Spinal Cord Stimulation Using Fluorodeoxyglucose Positron Emission Tomography. *Neuromodulation Technol. Neural Interface* **2019**, *23*, 56–63, doi:10.1111/ner.12960.
